# A Genome-Wide Hybrid Incompatibility Landscape between *Caenorhabditis briggsae* and *C. nigoni*

**DOI:** 10.1371/journal.pgen.1004993

**Published:** 2015-02-18

**Authors:** Yu Bi, Xiaoliang Ren, Cheung Yan, Jiaofang Shao, Dongying Xie, Zhongying Zhao

**Affiliations:** 1 Department of Biology, Hong Kong Baptist University, Hong Kong, China; 2 State Key Laboratory of Environmental and Biological Analysis, Hong Kong Baptist University, Hong Kong, China; University of Wisconsin–Madison, UNITED STATES

## Abstract

Systematic characterization of ẖybrid incompatibility (HI) between related species remains the key to understanding speciation. The genetic basis of HI has been intensively studied in *Drosophila* species, but remains largely unknown in other species, including nematodes, which is mainly due to the lack of a sister species with which *C. elegans* can mate and produce viable progeny. The recent discovery of a *C. briggsae* sister species, *C. nigoni*, has opened up the possibility of dissecting the genetic basis of HI in nematode species. However, the paucity of dominant and visible marker prevents the efficient mapping of HI loci between the two species. To elucidate the genetic basis of speciation in nematode species, we first generated 96 chromosomally integrated GFP markers in the *C. briggsae* genome and mapped them into the defined locations by PCR and Next-Generation Sequencing (NGS). Aided by the marker, we backcrossed the GFP-associated *C. briggsae* genomic fragments into *C. nigoni* for at least 15 generations and produced 111 independent introgressions. The introgression fragments cover most of the *C. briggsae* genome. We finally dissected the patterns of HI by scoring the embryonic lethality, larval arrest, sex ratio and male sterility for each introgression line, through which we identified pervasive HI loci and produced a genome-wide landscape of HI between the two nematode species, the first of its type for any non-*Drosophila* species. The HI data not only provided insights into the genetic basis of speciation, but also established a framework for the possible cloning of HI loci between the two nematode species. Furthermore, the data on hybrids confirmed Haldane’s rule and suggested the presence of a large X effect in terms of fertility between the two species. Importantly, this work opens a new avenue for studying speciation genetics between nematode species and allows parallel comparison of the HI with that in *Drosophila* and other species.

## Author Summary

Hybrid incompatibility (HI) has been intensively studied among *Drosophila* species, but remains largely unknown in other species. Model organism is a species of choice for the HI study because these species provide sophisticated molecular and genetic tools for illustrating mechanism underlying a given HI. *C. elegans* as a model organism contributed little to the field due to the lack of a sister species with which it can mate and produce viable progeny. The recent discovery of a *C. briggsae* sister species, *C. nigoni*, has made it possible to study the HI between nematode species. However, the paucity of molecular and genetic tools in both species inhibits their use in such studies. To empower the use of this species pair in HI studies, we first created a collection of fluorescent markers over the *C. briggsae* genome to facilitate the directional introduction of the marker-associated *C. briggsae* genomic fragments into the *C. nigoni* background. We next mapped the marker insertion sites and introduced the markers into *C. nigoni* by repeated crossings. Finally, we generated a genome-wide HI landscape between the two species by scoring the HI phenotypes of their hybrid progeny. The study not only provides an invaluable resource for the molecular cloning of HI loci between *C. briggsae* and *C. nigoni*, but also permits comparative analysis of speciation genetics between nematode and other species.

## Introduction

It is well known that many closely related species are able to mate with each other, but their hybrid progeny frequently die, become sterile or develop abnormally. This phenomenon is referred to as post-zygotic hybrid incompatibility, hereafter termed as HI. Mechanistic research into HI lies at the heart of biology because HI represents one of the most fundamental biological processes for limiting gene flow between species, which leads to the tremendous biodiversity encountered in daily life. The models independently proposed by Bateson, Dobzhansky and Muller, (now collectively called the BDM model), have been widely used to elucidate the genetic basis of HI. According to this model, HI involves incompatible epistatic interactions between multiple genes that are functionally divergent between the parental species [1, 2]. The predictive power of the BDM model has gained wide support from studies on the hybrids of various species [3, 4], although controversy exists over the methods used to evaluate the HI phenotypes [5]. Two empirical rules have been proposed to explain post-zygotic HI [6]. The first is called Haldane’s rule, which posits that the hybrid progeny of heterogametic sex are more likely to suffer sterility or lethality than those of homogametic sex. Two factors are believed to underlie this phenomenon. First, if the allele that causes HI is recessive, then the heterogametic hybrid progeny will manifest its full effects due to hemizygosity, whereas the homogametic hybrid progeny will not show such effects due to the compensation by a second copy of a wild-type allele. This explanation, dubbed the dominance theory [7], has gained support from genetic studies of HI in both animal and plant species. The second factor is that, hybrid male sterile loci may accumulate faster than hybrid inviable or female sterile loci, because male-specific genes are thought to evolve faster as a consequence of sexual selection. This idea is referred to as the fast-male theory [8]. In addition, analysis of gene expression in *Drosophila* species has suggested that the divergence of X-linked genes appears to be faster than that of autosome-linked genes [9]. This concept is called the fast-X theory. However, the theory has been challenged by results from comparative analysis of DNA sequences of different *Drosophila* species [10]. The second empirical rule proposed to explain HI is called the large X effect, which states that the substitution of X chromosomal fragments produces more severe HI than the similar substitution of autosomes. The large X effect has been systematically tested and confirmed in *Drosophila* species [11]. Whether the large X effect applies in other species is largely unknown.

By taking advantage of their abundant genetic and molecular tools, a few model organisms, especially *Drosophila* species, have been utilized to study the genetic basis of HI [12]. Such studies have provided unprecedented insights into how an intact genome ensures the faithful development of tissues and organs within a single species. However, *C. elegans* has contributed little to the field as a genetic model, primarily due to its lack of a sister species with which it can mate and produce viable progeny [13–15]. A pair of genes responsible for intraspecific HI has been identified between the different strains of *C. elegans* [16], but the genetics of interspecific HI is largely unknown between any nematode species. Global efforts in sampling *C. elegans* sister species have not been successful so far, but these efforts have isolated a sister species called *C. nigoni* for another nematode species, *C. briggsae* [17]. The sister species pair of *C. briggsae* and *C. nigoni* provides an opportunity to systematically map the HI loci between nematode species for the first time.

*C. briggsae* is related to *C. elegans* and its genome has been sequenced [18]. A handful of genetic tools have been developed for the species over the past decade, including those for efficient generation and mapping of chromosomally integrated transgene [19–21]. A *C. nigoni* draft genome is also available and under active annotation (Erich Schwarz, personal communication). Importantly, nematode species represent a very different taxon from those that have been intensively used for the study of speciation genetics, and the genes known to be responsible for any given HI phenotype show little conservation. Therefore, isolating the genetic loci of HI between nematode species holds promise for providing novel insights into speciation genetics. In *Drosophila* species, the mapping of HI loci has been achieved primarily by a combination of a P element transposon with a dominant and visible white gene as a marker [11]. The transposon allows random insertion of a transgene into a *Drosophila* genome while the white gene serves as a visible and semi-dominant marker that permits the selection of heterozygous transgenic animals out of those without transgene insertion. These tools greatly facilitate the use of *Drosophila* species as a popular model for the characterization of HI. However, a paucity of such tools in both *C. briggsae* and *C. nigoni* prevents them from being effectively used in similar studies. In an attempt to dissect genetic control of hermaphroditism, Woodruff et al. carried out crossings between the two species in both directions. Their data on the hybrids complied with Haldane’s rule in a cross-direction dependent manner. They also demonstrated a *C. briggsae* specific segregation distortion in the hybrids using a bulk segregant assay with 22 SNPs and one indel marker across the genome [17]. To characterize the strain dependency of HI between *C. briggsae* and *C. nigoni*, Kozlowska et al. performed reciprocal crossings between eight isogenic strains of *C. briggsae* and five inbreeding *C. nigoni* strains or one of its wild isolate. The results showed strain-dependent HI patterns in F1 hybrids, which again confirmed Haldane’s rule and demonstrated parent-of-origin HI effect [22]. However, a defined genome-wide landscape of HI between the two nematode species remains to be seen.

To facilitate the systematic mapping of HI loci between *C. briggsae* and *C. nigoni*, we first used biolistic bombardment to generate a large collection of independent chromosomally integrated transgenes, which are fusions of the *myo-2* promoter with GFP, (hereafter termed as myo-2::GFP). The transgene was used as a visible marker in the *C. briggsae* genome. We next mapped the GFP insertion sites into a defined genomic region using a method that we developed previously [19]. We finally dissected the genome-wide HI by repeatedly backcrossing the GFP associated *C. briggsae* into an otherwise *C. nigoni* background followed by phenotypic scoring. We then used the HI phenotypes to test the “two empirical rules of speciation” [6, 11].

## Results

### A high-density *C. briggsae* physical map was generated that was comprised of 96 chromosomally integrated fluorescent markers

**Fig 1:**
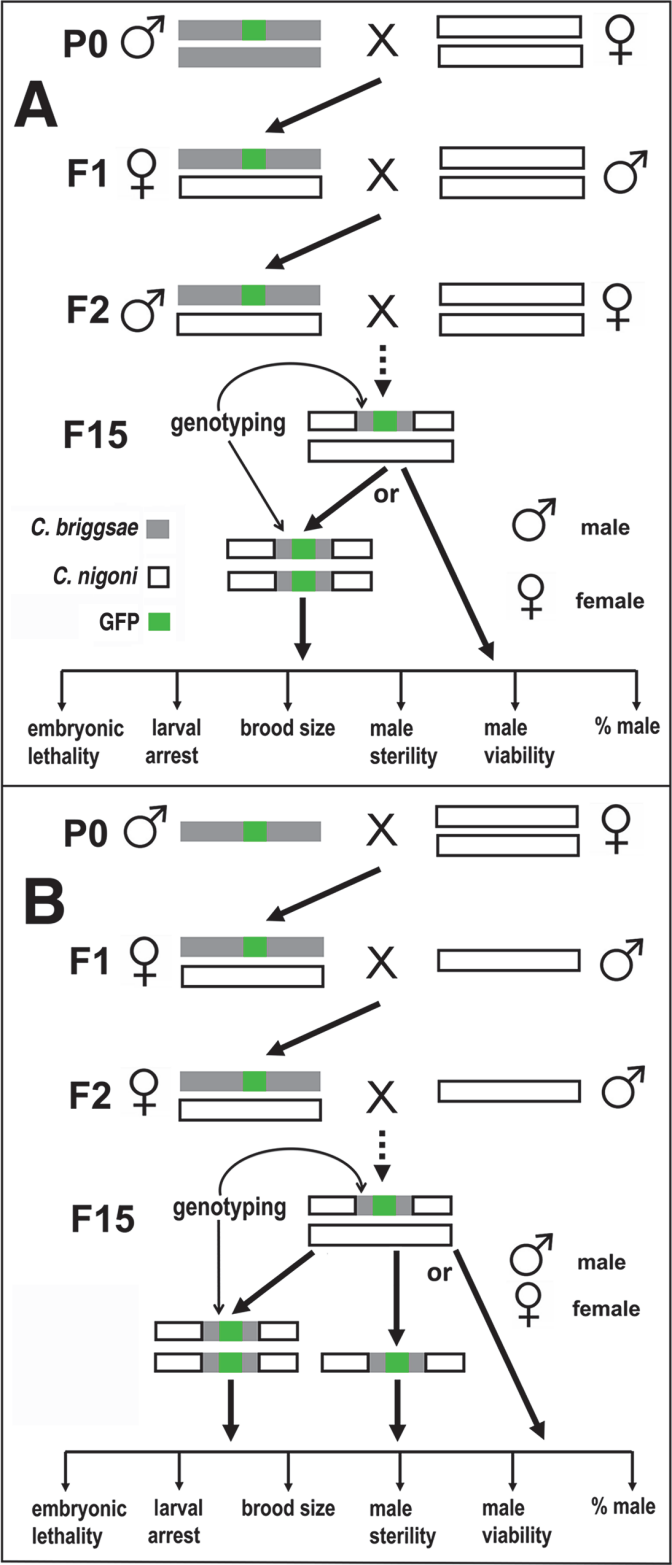
Introgression strategy. (A) Strategy for autosome-linked introgression. Introgression was initiated by crossing GFP-expressing *C. briggsae* males (in AF16 background) with *C. nigoni* (JU1421) virgin (L4 stage) females. The GFP-expressing F1 L4 females were then backcrossed with *C. nigoni* males followed by repeated backcrossing between F2 GFP-expressing males with *C. nigoni* L4 females. (B) Strategy for X chromosome-linked introgression. Introgression was initiated in the similar way as that for the autosome-linked introgressions, but only GFP-expressing L4 female progeny were used to backcross with *C. nigoni* males from the F2 generation onward. All of the introgressions were performed for at least 15 generations followed by genotyping with single-worm PCR. The HI phenotypes scored for homozygous or heterozygous introgressions are listed at the bottom of each panel (see Materials and Methods).

To empower the *C. briggsae* and *C. nigoni* species pair as a model for the dissection of HI loci, we created a physical map consisting of 96 stable transgenic markers of myo-2::GFP in *C. briggsae*. We used biolistic bombardment to generate chromosomally integrated *C. briggsae* transgenic lines that brightly expressed the markers in the pharynx (S1 Fig.). We produced a total of 97 independent stable transgenic strains expressing the GFP (S1 Table). All of the strains carrying a homozygous or hemizygous transgene were viable, without obvious defects in fitness, except for one strain that demonstrated a smaller brood size (fewer than 25) than that of the wild-type. This strain was excluded from the subsequent analysis. We first attempted to map the GFP insertion site through inverse PCR using the primers specific to the GFP vector. Unfortunately, the sequencing results revealed that all of the PCR products were derived from the vector itself, presumably because the transgenes produced by biolistic bombardment were present as tandem copies in the host genome. We then mapped the marker insertion sites following the method we developed previously [19] (Fig. 1 and S1 Table). Briefly, we first performed introgression for all of the independent GFP-linked *C. briggsae* genomic fragments into *C. nigoni* for at least 15 generations and made a total of 111 independent introgression lines (S1 Table). Consistent with the previous crossing results between the two species [17, 22], the lack of F1 fertile males supported Haldane’s rule. We then mapped the boundaries of the introgression fragments by PCR genotyping of the GFP-expressing animals using *C. briggsae* (cb4) [23] specific primers (S2 Table). These boundaries were used to estimate both the introgression size and the GFP insertion site in *C. briggsae* genome as described below. We validated the PCR-based genotyping results for eight out of the 13 homozygous introgression fragments using NGS (see Materials and Methods). The results from the two methods agreed well (S2 Fig., S3 Table), indicating that our PCR-based mapping method could reliably detect the introgression boundaries albeit with a lower resolution than the NGS method. Notably, the NGS sequencing recovered no *C. briggsae* genomic sequences (except for ZZY10291) other than those linked with GFP in the introgression strains (S2D–S2F Fig., S3 Table). We re-genotyped the boundaries of the introgression with PCR after another 60 generations of backcrossing, but the ambiguity persisted, as there was no change in the introgression size. This result raised the possibility that the fragment that was separate from the GFP marker but left in the *C. nigoni* background after the backcrossing was not due to the insufficient backcrossing, but was owing to a genome assembly error that placed the two fragments into separate parts of the chromosome II that actually belonged to a single chromosomal region. Consistent with this, the boundaries of the two fragments consisted of highly repetitive sequences, which could be responsible for the assembly error. In addition, we noticed another two genomic fragments associated with genotyping primers, X-4 and II-7.5 (S2 Table) respectively, which appeared to be placed into incorrect genomic positions according to our PCR-based genotyping data (S3 Fig.). For example, the PCR product of the X-4 primer was placed into an interval of chrII: 15,286,436.15,286,841 in the *C. briggsae* assembly “cb4”. However, the genotyping results for the introgression ZZY10353 suggested that it was probably placed there due to an assembly error, because all of its flanking primers showed the expected amplification except for the primer itself (S3 Fig.). Genotyping results from the introgression ZZY10320 supported it was located between the primer X-3.5 and X-4.5 on the chromosome X. Further genotyping with this primer would cause uncertainty if its position (annotated in “cb4”) was assumed to be correct. Therefore, we assigned introgression boundaries based on the consensus of genotyping results derived from multiple primers and did not count the primers that gave an exceptional result. A similar situation was observed for the primer II-7.5 (S3D Fig.). Taken together, 15 generations of backcrossing was sufficient to get rid of the sequences that were unlinked with the GFP markers, but the potential *C. briggsae* genome assembly errors may have complicated the interpretation of some mapping results.

**Fig 2:**
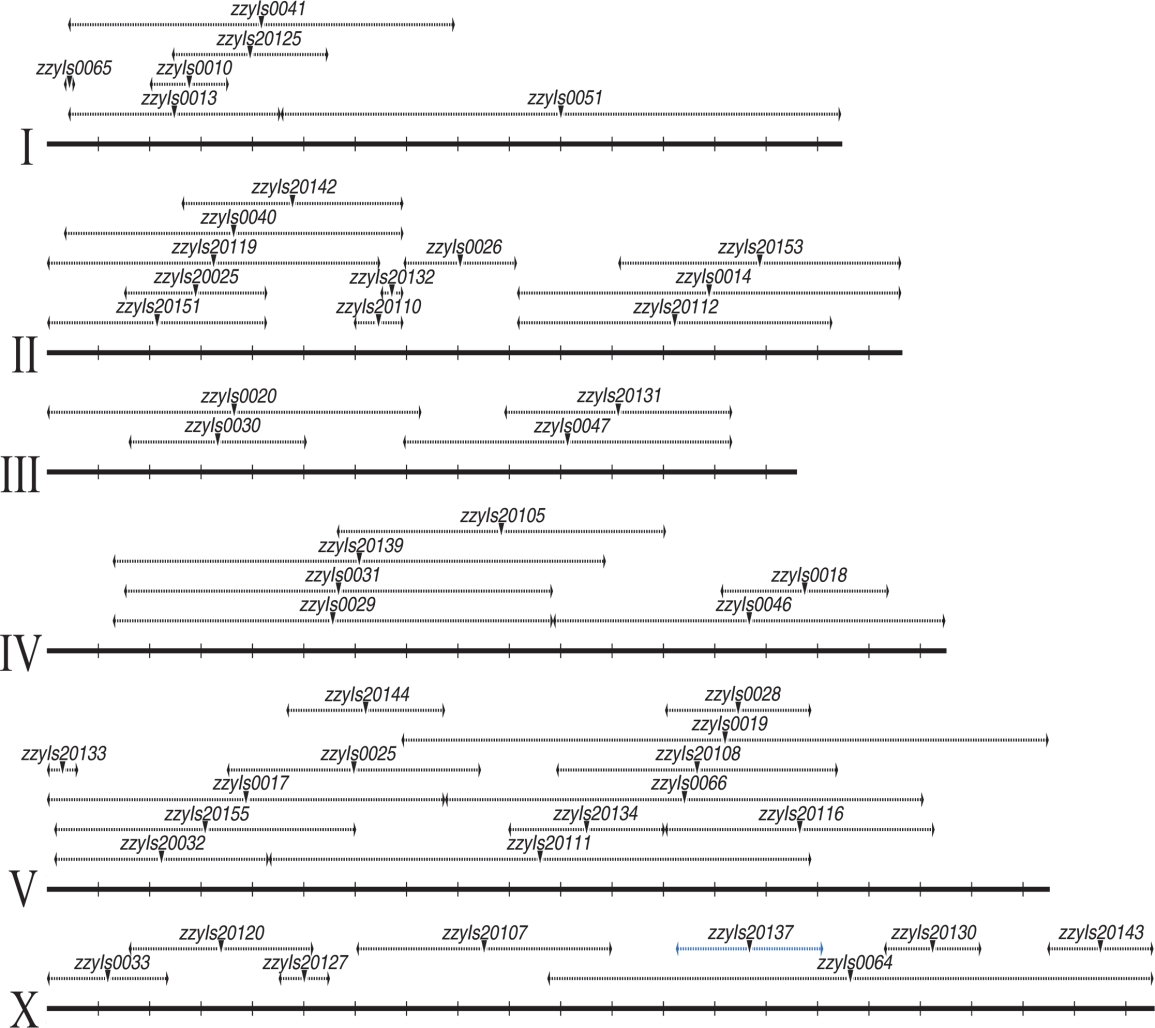
A *C. briggsae* physical map consisting of a subset of 48 chromosomally integrated transgenic GFP markers. Chromosome and mapped position for transgenic marker are depicted in scale as solid and dashed horizontal lines respectively based on the *C. briggsae* “cb4” genome assembly. Mid-point of the mapped transgene is indicated with an inverted triangle. Name of the transgene is indicated above. All the mapped boundaries are derived from a single transgene except for the transgene *zzyIs20137* (highlighted in blue) for which the boundaries were calculated from the overlapping regions of the two independent introgressions. Chromosome numbers are indicated on the left. Chromosomal coordinates are labeled at 1 Mb interval with vertical bars.

We produced a physical map of *C. briggsae* by using a subset of 49 independent mapped introgressions covering 48 independent transgenes as a proxy for the GFP insertion site (Fig. 2). We prioritized the subset based on the following criteria. First, the introgressions that were included were relatively small in size. Second, if multiple independent introgression lines were achieved for a single transgene, the overlapping regions between independent introgressions were used as the final introgression region for the transgene in the map (S1 Table). Finally, those transgenes with mapping complications associated with possible assembly errors were excluded in making the map. The large collection of the mapped GFP insertions and introgression lines paved the way for a systematic isolation of the HI loci between *C. briggsae* and *C. nigoni*, which has not been attempted between any nematode species.

### A large portion of the *C. briggsae* genome appeared difficult to recombine with *C. nigoni* chromosomes

**Fig 3:**
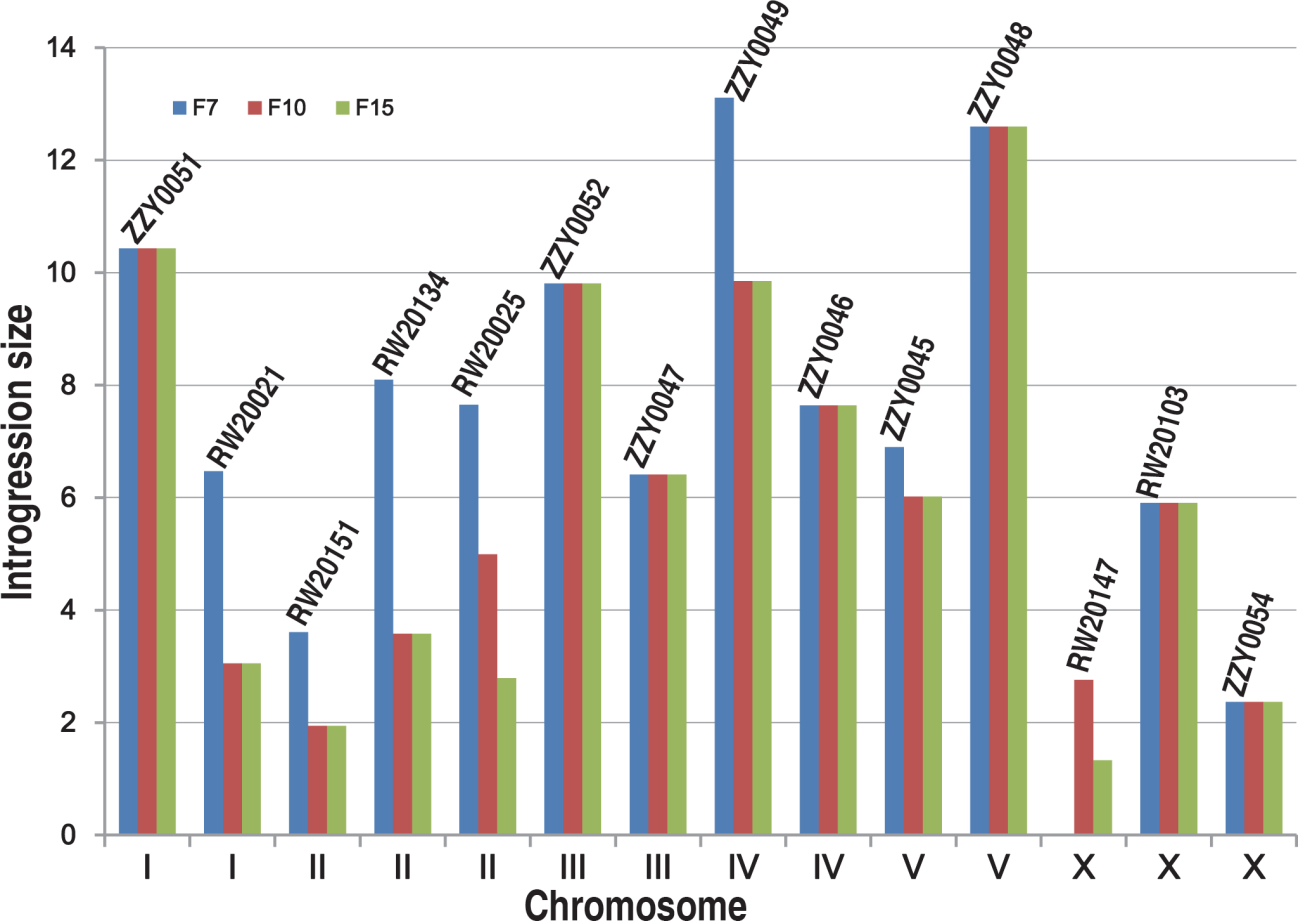
Effect of the number of backcrossing generation on the introgression size. Shown are changes in introgression sizes over backcrossing generations which are derived from 14 different transgenic strains with names indicated above. The boundaries of introgressions are genotyped immediately after 7^th^, 10th and 15^th^ generation of backcrossing. X and Y axis indicates *C. briggsae* chromosome numbers and introgression sizes respectively.

**Fig 4:**
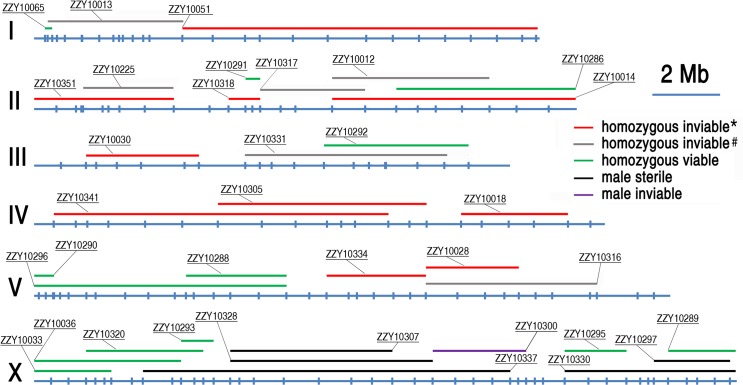
A genome-wide hybrid incompatible landscape between *C. briggsae* and *C. nigoni*. Individual introgressions (indicated by its strain name) are drawn in scale as horizontal bars above their source *C. briggsae* chromosomes (blue horizontal lines with the identity indicated on the left). The introgressions are differentially color coded according to their observed HI phenotypes as indicated when present as a homozygote or hemizygote in the *C. nigoni* background. Positions of the PCR primers used for genotyping are indicated in scale as small vertical blue bars. Only the introgressions that were selected for rendering homozygous are shown. *All HI phenotypes including sterility, viability, Emb, Lva and brood size were scored; # only viability and sterility were scored.

**Fig 5:**
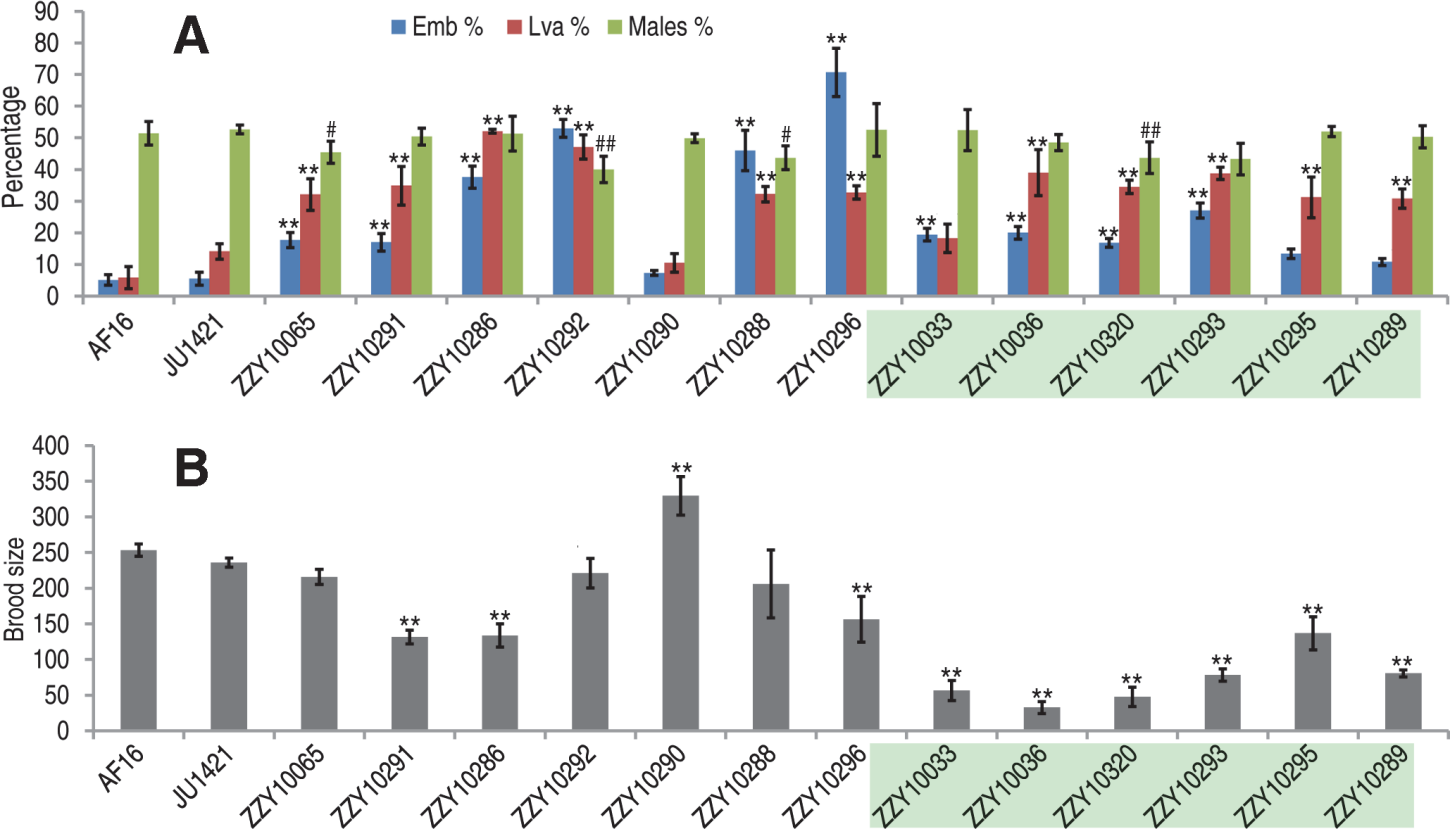
Hybrid incompatible phenotypes for the homozygous introgressions between *C. briggsae* and *C. nigoni*. (A) Shown are the mean percentages of Emb, Lva and male out of the total progeny for the homozygous introgressions. (B) Shown are the mean brood sizes for the homozygous introgressions. Names of the strains used for phenotypic scoring are indicated at the bottom. The names of the strains carrying an X-linked introgression are shaded. Emb, embryonic lethality; Lva: larval arrest; percentage of male progeny is calculated as the ratio of males out of total hybrid progeny (see Materials and Methods). Standard deviations (SD) are indicated as error bars. Statistical significances from comparing mean of Emb, Lva and brood size are indicated with “*” or “**” which denotes p<0.05 and p<0.01 respectively (One-way ANOVA followed by post-hoc test); “#” and “##” indicate p<0.05 and p<0.01 respectively in X^2^ test with an expected percentage of 50%. Sample size tested was listed in S4 Table.

Our initial plan was to characterize the HI, mainly by using homozygous introgressions. However, a large portion of the *C. briggsae* genomic regions seemed resistant to recombination with its homologous regions in *C. nigoni*, as judged by its introgression sizes over backcrossing generations (Fig. 3). Therefore, we attempted to render homozygous a subset of the 111 introgressions that were prioritized based on the following criteria. First, they were relatively small in size and showed minimal overlapping between one another. Second, the ratio of overall GFP-expressing progeny to the total hybrid progeny was as close as possible to 75% for a cross between parents that were both heterozygous for the GFP locus on the autosome. This criterion was chosen because if the ratio was close to 75%, then the animals carrying the homozygous introgressions were more likely to be viable, as was expected from the dominance feature of the GFP. We made such an attempt for a total of 35 independent introgressions (Fig. 4, S1 Table), of which 13 were successfully rendered homozygous, which represented 26.5% of the *C. briggsae* genome (Table 1, S4 Table, Fig. 5). The remaining 22 introgressions could not be rendered homozygous after five attempts, suggesting that they were inviable as homozygotes or sterile/inviable as hemizygotes (Fig. 4, S1 Table). Consistent with this finding, the recombination frequency between the two species was relatively low compared to that between *Drosophila* sister species. First, we found that the average sizes of the introgressions were far bigger than those of *Drosophila* species. For example, after 15 generation of backcrossing, the average sizes of the homozygous introgressions were approximately 3.2 and 2.6 Mb for the autosomal and the X chromosomal introgressions, respectively (S4C Fig.). However, the autosomal and X chromosomal introgressions between *D. mauritiana* and *D. sechellia* were reported to be approximately 1.3 and 1.0 Mb respectively [11]. The average sizes of the inviable introgressions were 4.6 and 7.7 Mb for autosomal and X chromosomal introgressions respectively between *C. briggsae* and *C. nigoni* (S5 Fig.) while those for the similar introgressions between *D. mauritiana* and *D. sechellia* were approximately 1.5 and 1.7 Mb, respectively. A second finding was that the benefit for reducing introgression sizes was limited in most strains after 10 generations of backcrossing. For example, the sizes of the introgressions from the transgenic line ZZY0051 and ZZY0048 were over 10 Mb and remained unchanged from the 7^th^ to the 15^th^ generations of backcrossing (Fig. 3). Among the 14 introgressions for which we tested the effect of backcrossing generations on the introgression size, seven showed no further recombination after the 7th backcrossing. Only two out of the 14 introgressions benefited from further recombination from backcrossing beyond 10 generations. In agreement with this, many inviable introgressions tended to be larger than those that were successfully rendered as viable homozygotes, which may partially explain why they failed to be rendered homozygous.

**Table 1:** Coverage statistics for introgressions along with its homozygous viability based on the “cb4” assembly of *C. briggsae* genome.

Linkage group	Chromosome size (Mb)	Total introgression size* (Mb)	Total coverage%	Total homozygous introgression (Mb)	Total homozygous introgression %	Total homozygousinviable introgressions (Mb)	Total homozygous inviable introgression%	Total heterozygous introgression size (Mb)	Total heterozygous introgression %
ChrI	15.45	15.13	97.93%	0.22	1.42%	14.91	96.50%	14.91	96.50%
ChrII	16.62	13.54	81.42%	5.96	35.84%	7.58	78.71%	13.54	81.42%
ChrIII	14.57	10.33	70.90%	4.44	30.47%	5.89	40.43%	10.33	70.90%
ChrIV	17.48	14.73	84.27%	0	0.00%	14.73	84.27%	14.73	84.27%
ChrV	19.49	13.65	70.04%	7.75	39.76%	5.90	30.27%	5.9	30.27%
Autosomal	83.62	67.38	80.58%	18.37	21.97%	49.01	58.61%	59.41	71.05%
chrX	21.54	20.36	94.52%	9.48	44.01%	10.88	50.51%	17.02	79.02%
Overall	105.16	87.73	83.39%	27.85	26.49%	59.89	56.96%	76.43	72.68%

* Redundant parts are consolidated in calculation of the size and percentage of all introgression.

### Phenotypic characterization demonstrated pervasive HI in the hybrids containing either homozygous or heterozygous introgressions

**Fig 6:**
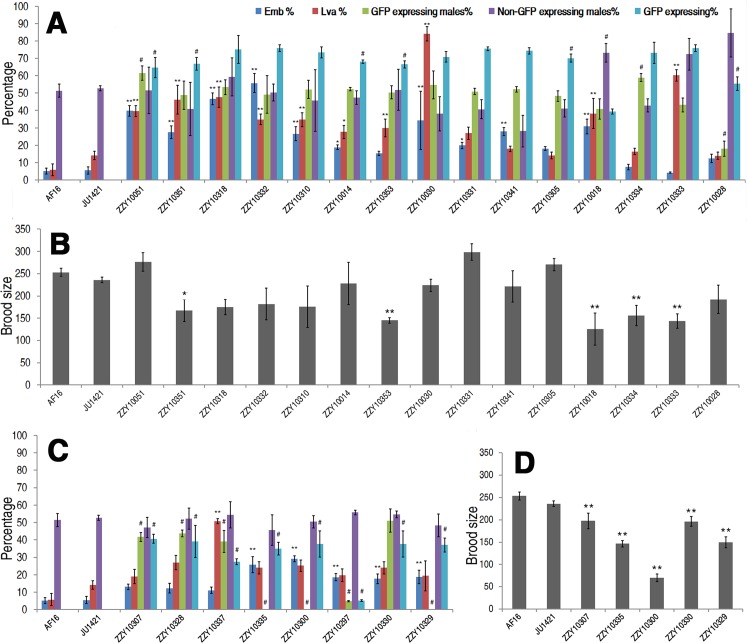
Hybrid incompatible phenotypes for autosome- or X-linked heterozygous introgressions between *C. briggsae* and *C. nigoni*. (A & C) Shown are the mean percentages of Emb, Lva, GFP-expressing or non-GFP-expressing male and overall GFP expressing progeny out of the total progeny for autosome- or X chromosome-linked introgressions respectively. (B & D) Shown are the mean brood sizes for the autosome- and X chromosome-linked introgressions respectively. Names of the strains used for phenotypic scoring are indicated at the bottom. Percentage of GFP expressing males is calculated as the ratio of GFP expressing males out of total GFP expressing hybrid progeny in a cross between parents both heterozygous for the introgression (see Materials and Methods). In the case of male sterile or inviable introgression, the percentage was scored in a cross between a JU1421 male and a female heterozygous for the introgression. Overall percentage of the GFP expressing progeny (GFP expressing %) is calculated the ratio of GFP expressing animals out of the total hybrid progeny. Statistical significances and standard deviations are labeled in the same way as that in Fig. 5. Sample size tested was listed in S5 Table.

To systematically score the HI phenotypes associated with individual introgressions, we characterized the HI phenotypes for all 13 homozygous and another 23 heterozygous introgressions, including most of the 22 inviable introgressions described above (S6 Fig.), and a few others that were not tested for homozygous viability (Fig. 4 and 5, S1 Table). We used fertility interchangeably with brood size, (which refers to the total number of eggs during the lifetime of an animal). The results demonstrated extensive HI for homozygous or heterozygous introgressions (Fig. 5 and 6, S4 and S5 Table). For example, the percentages of embryonic lethality and larval arrest for most of the homozygous introgressions were significantly higher than those for the *C. nigoni* control strain (JU1421) (post-hoc test after one-way ANOVA) (Fig. 5A). Brood sizes for nine out of the 13 introgressions were significantly smaller than that of the control (JU1421) (Fig. 5B). Intriguingly, the homozygous introgression ZZY10290 not only had little effect on embryonic and larval viabilities, but also led to a significantly bigger brood size than the control (JU1421) (Fig. 5B). A subset of the homozygous introgression strains, for example ZZY10296, were difficult to be maintained as lines, due to the high incidence of embryonic lethality and larval arrest (Fig. 5A), suggesting some lethal interactions between the genes within the introgression fragments and those of *C. nigoni*, although some interactions not be fully penetrant. Further evidence for the homozygous inviability of the autosomal introgressions came from an observation that the percentage of overall GFP-expressing progeny was significantly lower than the expected 75% for about half of the autosomal introgressions that we attempted to render homozygous (p<0.01, X^2^ test) (Fig. 6A, S5 Table, also see Materials and Methods). Surprisingly, over 44% of the *C. briggsae* X chromosomes could be present in *C. nigoni* as viable homozygous introgressions, whereas fewer than 22% of the autosomes were successfully made homozygous in our assay (Table 1). Again, given the complications associated with large introgression size, further analysis is needed to precisely dissect the number of HI loci involved in the lethal gene interactions between the two species. In contrast to the fertility and fitness results, most of the homozygous introgressions did not produce significant deviations in male ratio, regardless of the introgressions located on the autosome or on the X chromosome (Fig. 5A).

In addition to the overall ratios of GFP-expressing progeny to the total hybrid progeny, other HIs were frequently observed for many heterozygous introgressions. For example, 15 out of the 23 heterozygous introgressions resulted in significantly higher ratios of embryonic lethality and three of the autosomal introgressions, ZZY10051, ZZY10318 and ZZY10332, appeared to be at least partially dominant in terms of embryonic lethality (Fig. 6A). We considered an autosomal heterozygous introgression to have a dominant effect if the embryonic lethality ratio of the hybrid progeny (from a cross between parents that were both heterozygous for the introgression) was higher than the sum of the lethality ratio of the control and 25% (which was the expected ratio of homozygous introgressions, assuming that all of the embryos were dead). A significantly higher ratio of larval arrest was observed in 11 out of the 23 heterozygous introgressions, two of which, ZZY10030 and ZZY10333, appeared to be dominant (Fig. 6A). Most of the heterozygous introgressions showed significant reductions, whereas three of them, ZZY10051, ZZY10331 and ZZY10305, demonstrated apparent elevations in fertility (Fig. 6B). Further evidence of HI came from the differential ratio of GFP-expressing male to female hybrid progeny from crossing between two parents that were heterozygous for the GFP locus. This is because 75% of the male hybrid progeny from autosomal introgressions were expected to express GFP, and 50% were expected to express GFP from X chromosomal introgressions, provided that all of the introgression-containing males were viable. Seven out of the eight heterozygous X chromosomal introgressions demonstrated a significant reduction in the ratio of GFP-expressing males in the hybrid progeny, whereas only three out of the fifteen heterozygous autosomal introgressions showed a similar deviation. This result was probably due to the dominance effect of the introgression, which was manifested in the hemizygote males for the X-linked introgressions, but was masked in the heterozygote males for the autosome-linked introgressions.

Surprisingly, two of the three autosomal introgression-containing strains, ZZY10051 and ZZY10334, showed significant elevations in the ratios of GFP-expressing males, whereas only a single autosomal introgression-containing strain, ZZY10028 demonstrated a significant decrease in the ratio (p<0.01, X^2^ test) (Fig. 6A). For X chromosomal introgressions that produced male sterility or inviability, backcrossing was performed in the opposite direction relative to that used for the autosome-linked introgressions. In other words, a GFP-expressing female was crossed with a *C. nigoni* male. Therefore, if all of the GFP-expressing males were viable, 50% of the male crossing progeny were expected to express GFP. However, if all of the GFP-expressing males were inviable, then 33.3% of the total crossing progeny were expected to express GFP. A significant deviation from the expected ratio, (also called a distortion of sex segregation), would suggest sex-specific embryonic lethality or larval arrest that could be caused by homozygous, heterozygous or hemizygous introgressions. Intriguingly, less than half of the autosomal heterozygous introgressions produced hybrid progeny that expressed GFP at a ratio significantly lower than the expected 75% of the total progeny. However, the GFP-expressing ratio for all of the X chromosomal heterozygous introgressions were significantly lower than the expected 50% and 33.3% for male viable and inviable introgressions, respectively (p<0.01, X^2^ test) (Fig. 6A and C). Whether this result reflected the large X effect remains to be determined, due to the complications associated with introgression sizes (S5 Fig.) and the different crossing strategies used for male sterile and male fertile introgressions.

### Hybrid male sterile and male inviable loci were frequently observed in the middle and right arm of the X chromosome

**Fig 7:**
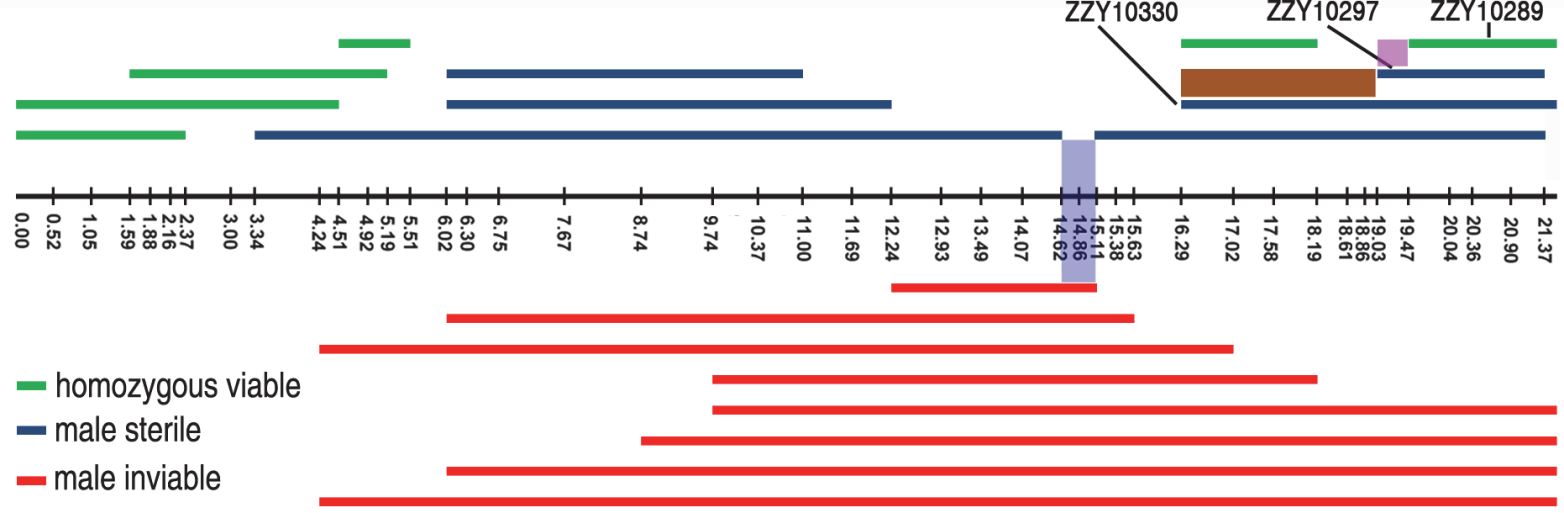
Refined mapping of HI loci on X chromosome by contrasting the HI phenotypes between independent introgressions. Male fertile (green), male sterile (blue) and male inviable introgressions (red) are depicted along the X chromosome, which is drawn as a horizontal black line with positions of the genotyping primers indicated by black vertical bars. The corresponding chromosomal coordinates in Mb are indicated below the bars. Small intervals mapped for male viability and male sterility are shaded in blue and pink respectively. The region containing a potential suppressor of male viability is shaded in brown. The strain names for a subset of introgressions are indicated.

Our X-chromosome linked GFP markers allowed systematic isolation of male sterile or male inviable loci due to their full penetrance as X-linked introgressions. For an X-linked introgression, male inviability was defined as the absence of any GFP-expressing F1 male progeny, but the presence of GFP-expressing female progeny in a cross between a GFP-expressing virgin female (L4 staged female) and a *C. nigoni* young male. Similarly, male sterility was defined as the absence of any F1 progeny in a cross between a male bearing an introgression and a *C. nigoni* virgin female. We obtained a total of 18 independent transgenic strains with transgene insertions located on various parts of the X chromosome (S1 Table). We successfully rendered homozygous six X-linked introgressions in the *C. nigoni* background, which occupied 44.0% of the *C. briggsae* X chromosome (Fig. 7 and Table 1). Intriguingly, all of the homozygous viable introgressions were located on both arms of the X chromosome, whereas the multiple male sterile introgression (at least two) and the inviable introgressions (at least one) were mainly observed in the middle or right arm (Fig. 7).

By contrasting multiple overlapping introgressions, it could be deduced that there were at least two male sterile loci (one in the middle and the other on the right arm of the X chromosome) and one inviable locus (Figs. 4 and 7). For example, by comparing the HI phenotypes of the X-linked inviable and sterile introgressions ZZY10337 and ZZY10300, one could infer that a chromosomal interval of roughly 490 kb in size (from 14.62 to 15.11 Mb) was essential for male viability (Fig. 7). It should be noted that the observed inviability may not necessarily be male specific, because the introgression was fully penetrant in the male, but may have been masked in the female as a heterozygote. Comparison of another two X-linked introgressions, ZZY10297 and ZZY10289, allowed us to deduce a male sterile locus within an interval of approximately 440 kb region (Fig. 7). Interestingly, comparison of the heterozygous overlapping introgressions between ZZY10297 and ZZY10330 revealed a sharp contrast in the percentages of GFP-expressing male progeny and all GFP-expressing progeny (Fig. 6C). Both of these introgressions were located on the right arm of the X chromosome. They were 2.34 and 5.25 Mb in size, respectively and they produced male sterility as hemizygote. The introgression ZZY10297 was a subset of the introgression ZZY10330 (Fig. 7). Unexpectedly, the former produced only 4.9%, while the latter yielded 51.1% GFP-expressing males. In addition, ZZY10297 produced only 3.4% GFP-expressing progeny, but ZZY10330 produced 37.6% GFP-expressing progeny. This result raised the possibility that there was a closely linked suppressor locus for male viability, probably located somewhere within the introgression ZZY10330, but away from the introgression ZZY10297. On the other hand, some of the male sterile and inviable loci appeared to be closely linked. For example, the introgression in the strain ZZY10297 was classified as male sterile, but its GFP-expressing male progeny demonstrated significantly lower viability than that of the control (JU1421) (p<0.01, X^2^ test) (Fig. 6C). Interestingly, once a male carrying an X-chromosomal introgression (or hemizygous introgression) was fertile, the introgression was successfully made homozygous (Figs. 4 and 7). The observation suggested that X-linked female sterile or inviable loci were rare.

In contrast to the situation on the X chromosome, male sterile or inviable loci appeared to be rare on the autosome-linked introgressions. First, we found that only one out of the seven autosomal homozygous introgressions produced a significantly lower ratio of male progeny (x^2^ test, p<0.01) (Fig. 5), suggesting that sex-specific inviability or gamete production was uncommon in homozygous autosomal introgressions. Second, we observed that among the progeny from most of the crosses between parents that were both heterozygous for the introgression, approximately 75% expressed GFP during our efforts to make homozygous introgressions (Fig. 6A). This ratio suggested that most of the GFP-expressing male progeny were viable regardless of the presence of introgressions as a homozygotes or heterozygotes. For example, if a homozygous autosomal introgression led to sterile male progeny, we would expect that the male F1 crossing progeny to have a 12.5% chance of being sterile during our efforts to make homozygous introgressions (Materials and Methods). However, we did not find any males that failed to produce progeny, which suggested that male sterility was rarely caused by homozygous autosomal introgression. Interestingly, two out of the 15 autosomal heterozygous introgressions produced a significantly higher percentage of GFP-expressing male progeny. Whether this reflected a shift in the ratio of X or O sperm production by GFP-expressing males or sex-specific inviability remains to be determined.

### *C. briggsae* introgression fragments functioned mostly as recessive loci in an otherwise *C. nigoni* background

All of the introgressions were able to propagate to their last introgression steps, at least as a heterozygote, and all of the males and females carrying a heterozygous introgression were fertile, indicating that few introgressions were dominant enough to produce complete inviability or sterility as a heterozygote. Therefore, most of the *C. briggsae* introgressions acted as recessive loci in a *C. nigoni* background. This pattern was striking because some of the introgressions were over 10 Mb in size. Consistent with previous studies [17, 22], GFP-expressing fertile males were rarely found in the F1 progeny, but were readily identified in the F2 progeny of all autosome-linked introgressions (Fig. 1). In contrast, GFP-expressing fertile females were easily spotted in the F1 progeny of all of the autosome-linked introgressions. These results supported the dominance theory of Haldane’s rule, which claims that alleles causing HIs are partially recessive [7]. Further support for the dominance theory came from the observation that approximately 57% of the *C. briggsae* genome was inviable as a homozygote in *C. nigoni*, but could be readily maintained as a heterozygote (Table 1). However, it is likely that the genes involved in male sterility are different from those involved in female sterility, which could account for some of the observed differences between the two sexes.

We observed a significant sex segregation distortion from the introgression of ZZY10028 that produced 18% GFP-expressing males and 82% GFP-expressing females. As the ratio of larval arrest was comparable between ZZY10028 and the control (JU1421), the observed difference in embryonic lethality could not fully account for the deviation in the sex ratio, suggesting that the introgression functioned as a dominant locus on the sex ratio. The distortion of the sex ratio might have been contributed by some unidentified factors affecting the segregation ratio of X and O sperm. To investigate this possibility, we counted the ratio of GFP-expressing females and the ratios of non-GFP-expressing males and females. Surprisingly, the reduction in GFP-expressing males appeared to be largely compensated by an increase in non-GFP-expressing males. In agreement with this observation, previous work in *C. elegans* demonstrated that the difference in autosome size interacted with X hemizygosity in male meiosis, leading to predictable segregation distortion effects. In other words, hermaphrodites tended to inherit the shorter chromosome while the males tended to inherit the longer chromosome [24]. Our *C. briggsae* introgression fragment was probably smaller in size than its syntenic region in *C. nigoni*, given its origin of a hermaphroditic species [25, 26]. Therefore, a *C. nigoni* chromosome containing the introgression was predicted to be smaller than its homologous chromosome without the introgression, resulting in a biased segregation of GFP-positive chromosomes in females but of GFP-negative chromosome in males. This pattern may have underlain the observed segregation distortion. For the X-linked introgressions, given the presence of both heterozygous and hemizygous introgressions, it is obvious that all of the male sterile and inviable loci functioned recessively because all of the females carrying the same heterozygous introgression were viable and fertile. Using a calculation method similar to that used for autosomal introgressions, we demonstrated that the heterozygous introgressions in ZZY10300 appeared to be dominant in terms of fertility (Fig. 6, S5 Table).

### Fertility data of homozygous introgressions were consistent with the rule of large X effect

Haldane’s rule has gained wide support from various hybrid studies [11, 17, 22], but the rule of large X effect has been evaluated in few species other than those of *Drosophila*. According to the large X effect rule, substitution between two related species on the X chromosome has a relatively larger effect than a similar substitution on the autosome [27]. We tested the rule by comparing the HI phenotypes between X-linked and autosome-linked homozygous introgressions. All of the X chromosomal introgressions resulted in a significantly greater decrease in fertility than that from the autosomal introgressions (p<0.01, post-doc ANOVA) (Fig. 5A and 5B). However, the mean size of the autosomal introgressions was slightly larger than that of the X-linked ones (S4C Fig.), which was consistent with the rule of large X effect. However, the remaining HI phenotypes of the homozygous introgressions, including embryonic lethality, larval arrest and the percentage of hybrid male progeny expressing GFP, demonstrated no significant changes between the X-linked and the autosomal introgressions (p>0.01, one-way ANOVA) (S7 Fig., Fig. 5). Therefore, only the fertility data from the homozygous introgressions appeared to be consistent with the large X effect. It is worth noting that some of the introgressions used for fertility analysis were not independent from each other, which may complicate the interpretation of the data.

To develop a better understanding of the male sterility caused by the introgressions, we examined the germline morphology for a male sterile strain, ZZY10307, that carried a relatively short X-linked introgression (Fig. 7, S1 Table). DAPI staining showed that the number of sperms produced by the sterile adult male was in sharp contrast to that produced by the *C. nigoni* male (S8 Fig.). In addition, the observed male sterility did not seem to be caused by defective mating, because a clear mating plug was present after mating ZZY10307 sterile males with *C. nigoni* L4 females, suggesting that defective spermatogenesis caused by the introgression was probably to blame for the sterility. We were unable to reliably detect apparent sperms in the mated female (S8 Fig.). Therefore, we were uncertain whether the sterility was caused by an insufficient number of sperms delivered to the female or by the poor quality of the sperms. Further studies are needed to distinguish these possibilities.

## Discussion

### A new avenue for studying inter-species hybrid incompatibilities between nematode species

HI genes and their loci have been isolated between many closely related species, but never between nematode species. As the isolated genes underlying various HI phenotypes demonstrated little conservation among species, understanding the mechanisms of HI relies on the identification of more causative loci, (preferably genes in different taxa), and on comparative analysis of how these loci disrupt the normal development of tissues and organs in the hybrid progeny. Unfortunately, it has been infeasible to use *C. elegans* as a genetic model organism for this purpose, due to the lack of a sister species with which it can mate and produce viable hybrid progeny [13]. The recent discovery of a sister species for *C. briggsae* raised the possibility of using the species pair in studying the genetic basis of reproductive isolation between nematode species for the first time [17]. *C. briggsae* has been developed as a companion species of *C. elegans* for comparative study over the past two decades. The two species diverged from their common ancestor about 30–100 million years ago [18, 28]. However, the lack of efficient molecular and genetic tools prevents *C. briggsae* from being used for the systematic mapping of HI loci. Therefore, previous studies of speciation genetics with the two species have not been able to map a HI phenotype to a defined genomic region [17, 22]. Despite this, Woodruff et al. managed to use SNP and the indel-based bulk segregant assay to demonstrate that 13 *C. briggsae* genomic fragments showed significant distortions in segregation [17]. The results of their study were largely consistent with our observations. For example, all of the markers on chromosome IV showed substantial distortions in segregation, which was consistent with our finding that none of our introgressions on chromosome IV could be rendered homozygous in a *C. nigoni* background (Fig. 4).

The large collection of chromosomally integrated GFP markers and their derived introgression lines generated in this study provide an entry point for molecular characterization of the genomic regions responsible for a given HI phenotype between the two nematode species. The methodology used here can be readily adapted to any other *Caenorhabditis* species. For example, in addition to the fertile hybrid progeny that can be produced between *C. briggsae* and *C. nigoni*, a recently isolated *Caenorhabditis* species, *C. latens*, was found to be able to mate with *C. remanei* and produce viable hybrid F1 progeny, although significant breakdown was observed in the F2 hybrid [15, 29]. The F2 breakdown was more dramatic in the hybrid progeny between *C. briggsae* and *C. nigoni*. For instance, crossing between an F1 hybrid male and a female of either *C. briggsae* or *C. nigoni* has not been successful [17]. Studying the genetic basis of reproductive isolation between *C. remanei* and *C. latens* might have some advantage, given that both are obligatory outcrossing species, and this would avoid the potential complications of different reproduction modes seen between *C. briggsae* and *C. nigoni*. Future work needs to establish similar tools in *C. remanei* and/or *C. latens* as well as in other nematode hybrid viable species by using the recently developed genome editing techniques [30–32] to enable definitive mapping of the HI loci.

### Possible explanations for male sterility caused by X-linked introgression

Previous expression analysis using microarray showed that *C. elegans* germline enriched genes were mostly located on the autosome, with few on the X chromosome [33]. This bias was particularly strong for spermatogenesis genes. Multiple lines of evidence support the hypothesis that the X-linked genes are mostly silenced in the germline [34–36]. Consistent with this, our sterile males appeared to develop defective spermatogenesis, raising the possibility that the introgression on the X chromosome could de-silence the X-linked genes in an otherwise *C. nigoni* background, which might be responsible for the defective spermatogenesis. Alternatively, the “fast-X” hypothesis claims a faster rate of divergence for X-linked genes than for autosomal genes. Although this hypothesis has gained supporting evidences from studies in *Drosophila* species [9, 37, 38], it remains controversial [10]. If this is the case, it is possible that many X chromosome-linked genes involved in spermatogenesis may have diverged too far to be able to functionally replace each other between the two species. Finally, male-specific genes are thought to diverge faster than other genes due to sexual selection, i.e., the so-called fast-male theory [8]. Distinguishing these possibilities will demand a high quality of genome for both *C. briggsae* and *C. nigoni*. The *C. briggsae* draft genome was released nearly 10 years ago [18] with a few updates on its assembly [23, 39], but apparently there are still numerous errors in both assemblies [19, 40]. We encountered quite a few uncertainties in our genotyping of introgressions (S1 and S2 Table, S3 Fig.), suggesting the possible assembly errors in the relevant genomic regions.

### Complications of large introgression sizes

Given the relatively large sizes of the introgressions compared to those of *Drosophila* species, we cannot be certain whether a single or multiple loci were responsible for an observed HI phenotype within a given introgression fragment. The large size of the introgressions could also explain the hybrid inviabilities frequently observed between the two species. There are a few plausible explanations for their large size. First, different reproduction modes between hermaphroditic *C. briggsae* and gonochoristic *C. nigoni* likely lead to a smaller genome size in the former than in the latter. Consistent with this, all the sequenced gonochoristic *Caenorhabditis* species have a bigger size of genome and transcriptome than that of the hermaphroditic ones [25, 26]. Preliminary assembly of *C. nigoni* genome also suggests its genome size is substantially bigger than that of *C. briggsae* (personal communication, Eric Haag and Barbara Meyer). Chromosome size disparities caused by insertion or deletion between *C. briggsae* and *C. nigoni* may impair synaptonemal complex formation during meiosis. Second, despite intensive inbreeding of the outcrossing strain of *C. nigoni* strain JU1421, a substantial portion of its genome may still be heterozygous as is the case in other outcrossing species *C. brenneri* and *C. remanei* [25]. This is because some of the deleterious alleles must be present as a heterozygote to be viable. If the reduced fitness in the hybrids is produced by haploinsufficiency and is polygenic, the alleles in its *C. briggsae* syntenic regions may not be able to complement the fitness reduction either due to the possible mis-regulation of its expression in *C. nigoni* background or rearrangement as described below. Third, the genetic divergences between the two species are substantial and there might be quite a few chromosomal rearrangements between the two genomes, which could inhibit recombination. In agreement with this, a recent nucleotide sequence comparison suggested that the two diverged from their common ancestor around one million years ago [41], while the *Drosophila* species used in similar HI studies shared a common ancestor around 300,000 years ago [11, 42]. Fourth, our gene transformation method may have introduced some balanced but cryptic chromosomal rearrangements in the *C. briggsae* genome because it involved a mechanical force to break the chromosomal DNA to integrate the transgene into the host chromosome. However, the chance of such rearrangement is unlikely to be high. On the one hand, the transgenic strains were subject to backcrossing for at least two generations before crossing with *C. nigoni*, through which many of the balanced chromosomal rearrangements would be removed. On the other hand, our mapping of introgression boundaries with NGS did not identify very few *C. briggsae* genomic fragments that were not linked with the GFP marker but present in the introgression animal. In addition, the chance of inducing balanced chromosome arrangements such as translocation by bombardment should be relatively low. Notably, the collection of the introgressions that are large in size and resistant to recombination can be used as a genetic balancer that would facilitate the other genetic study in *C. nigini*. Currently, GFP markers are only available in the *C. briggsae* genome, making it impossible to examine the hybrid incompatibilities by the crossing in the opposite direction even the parent-of-origin effect was observed the hybrid progeny between the two species [17, 22]. Given the new developments in genome manipulation using TALEN or CRISPR/Cas, the generation of such markers in *C. nigoni* is within reach. Alternatively, by taking genomic approach, one presumably could evaluate the recombination frequency between the two species and examine selection against homozygous or heterozygous introgressions in both directions by genotyping near isogenic lines (NIL).

It is intriguing that a few introgressions, either as homozygote or heterozygote, increased brood sizes, suggesting some outcrossing benefits possibly through suppressing the inbreeding depression in *C. nigoni*. In agreement with this, the percentages of both embryonic lethality and larval arrest were much higher in the *C. nigoni* inbreeding strain JU1421 than those of the *C. briggsae* laboratory strain ([22], Fig. 6). For example, JU1421 demonstrated 14.1% of larval arrest compared to 5.8% in the *C. briggsae* AF16 strain, which could confound the interpretation of an observed HI phenotype. In addition, previous study suggested that the reduced fitness of hybrid progeny was caused by multiple interactions between *C. briggsae* and *C. nigoni*, a phenomenon called polyfactorial interspecies incompatibility [17]. This phenomenon seems to be common in other species [43, 44]. Interaction between inbreeding depression loci and outbreeding incompatible loci may further complicate the interpretation of an observed HI phenotype between the two species. Given the error-prone feature of the single-worm PCR, the mapped transgene insertion sites/introgression sizes may not be error free even though the mappings were performed in parallel with both positive and negative controls. We would like to urge caution in using the introgression strains and recommend a rigorous check of the genotype of interest before embarking on any detailed genetic or molecular characterization. Potential *C. briggsae* genome assembly errors identified during our PCR-based or NGS-based mapping may complicate interpretation of mapping results. An improved *C. briggsae* genome assembly will be needed to resolve the ambiguity. In summary, our introgression data provide a preliminary landscape of hybrid incompatibilities between *C. briggsae* and *C. nigoni* and established a framework for further characterization of molecular identities underlying various HIs, especially those of male sterility and inviability between the two nematode species.

## Materials and Methods

### Strains and maintenance

All of the *C. briggsae* strains used were AF16 or its derivatives. *C. nigoni* strain JU1421, an inbred line of JU1325 [17], was used for all introgressions due to its relatively low level of inbreeding depression [22]. All strains were maintained on regular NGM plates seeded with food *E. coli* OP50 at 25°C but with a higher concentration of agar (1.5%).

### Generation of stable transgenic strains of *C. briggsae*

Stable transgenic lines of *C. briggsae* were generated as described previously [19]. A plasmid construct, pSO159 [45], was doubly digested by restriction enzyme SpeI and ApaI to remove the his-72 fragment and replace it with the *cbr-myo-2* promoter to give rise to construct pZZ0031. The promoter was amplified from *C. briggsae* genomic DNA with the primers cbr-myo-2-F: tgcccgtgttatcaattagag and cbr-myo-2-R:ttcgtgatcCATtgctgtgt. The *unc-119* rescuing fragment remained the same as that in pSO159. The construct was introduced into *cbr-unc-119* deletion mutant strain RW20000 [20] by biolistic bombardment. Transgenic strains showing 100% rescue and bright expression of myo-2::GFP under a fluorescence stereomicroscope were retained for subsequent mapping and introgression.

### Mapping of transgene insertion site by single-worm PCR

Mapping of the transgene insertion site was performed essentially as described [19]. Mapping was achieved by crossing the GFP-linked *C. briggsae* genomic fragment into *C. nigoni* for at least 15 generations followed by genotyping with single worm PCR using *C. briggsae* specific primers. The size of the introgression fragment was calculated as the interval between the two primers that did not produce any PCR product and lay next to the left-most and right-most primer pairs that gave rise to successful PCR amplifications. The introgression regions were used as a proxy for the GFP insertion site in the *C. briggsae* genome when making the physical map (Fig. 2). Positive (*C. briggsae* (AF16)) and negative (*C. nigoni* (JU1421)) control worms were performed in parallel in all PCR genotyping steps. As the *C. briggsae* genome assembly “cb4” became available after we published the mapping method, there were substantial changes in the genomic positions of the mapping primers compared to those in the “cb3” assembly. An updated list of primers and its genomic positions associated with “cb4” assembly was listed in S2 Table. In cases where a mapping result from a single genotyping primer pair is inconsistent with those using its multiple flanking primer pairs, only the genotyping results from the latter was retained as the final result while that from the single exception was highlighted in S1 and S2 Table.

### Mapping of the transgene insertion sites by NGS

To validate a subset of the mapping results obtained from single-worm PCR using an independent method, sequencing libraries for C. briggsae (AF16), C. nigoni (JU1421) and introgression strain were constructed using an Illumina Nextera® XT DNA Sample Preparation Kit. Approximately 4 million paired-end reads per sample were produced using the Illumina MiSeq platform. The reads were then aligned against the reference sequence which was a combination of the C. briggsae genome “cb4” and C. *nigoni* contigs (the latter were a gift from Erich Schwarz) using Bowtie 2 (version 2.1.0) [46] in “sensitive-local” mode to find the best alignment positions in the two genomes. The “bam” files containing the best aligned reads were converted to wiggle format using RSeQC [47], normalized by the total bases sequenced for each sample. The average coverage of the *C. briggsae* “cb4” genome was calculated in a 10-kb window and plotted against *C. briggsae* genome using the R package. The regions with a five-fold higher coverage than those of its flanking regions were assumed to be caused by insertion of the *C. briggsae* fragment into the C. *nigoni* genome.

### Introgression strategy

All the GFP labeled *C. briggsae* strains were backcrossed to AF16 at least twice before they were used for introgression. Given all the GFP markers were generated in *C. briggsae*, backcrossing of the GFP marker was limited to one direction, i.e., from *C. briggsae* to *C. nigoni*. Therefore, introgression was initiated by crossing seven GFP-expressing *C. briggsae* males with five virgin *C. nigoni* females (L4 stage JU1421). Five GFP-expressing F1 L4 females were crossed with seven *C. nigoni* males. Seven GFP-expressing F2 males were crossed with five *C. nigoni* L4 females for the autosome-linked introgression. From F3 onward, only a single GFP-expressing male was crossed with three *C. nigoni* L4 females with five replicates. The worms for the subsequent crossings were always picked from a single crossing plate. The crossing was reiterated for another 13 generations to produce an introgression line with at least 15 generations of introgression (Fig. 1). For an X-linked introgression, the initial crossing steps were the same as those of the autosome-linked introgression up to the F2 generation. However, X-linked introgression frequently led to male inviability or sterility, particularly during the first 10 generations of introgression. Thus, from the F2 generation onward, a single GFP-expressing L4 female was crossed with three *C. nigoni* males with five replicates for a total of 15 generations. However, for each transgene, due to large number of strains were handled simultaneously, only strains carrying independent introgressions derived from the same transgene but with different sizes were kept in frozen tank in most cases. Control introgression was conducted in parallel in five replicates using the same scheme by crossing *C. nigoni* males with *C. nigoni* L4 females (both are JU1421 worms).

### Generation of homozygous introgression

After 15 generations of introgression the GFP-expressing animals were de-contaminated by egg preparation. Seven young male adults and five L4 females, both heterozygous or hemizygous for GFP from progeny of the 15-generation introgression, were used as P0 and mated on a single NGM plate for two and a half days. From the F1 cross progeny, a single GFP-expressing young male (homo- or heterozygous) and a single GFP-expressing female L4 worm (homo- or heterozygous) were mated on an individual plate with 12 replicates (one out of nine plates is expected to be homozygous for both male and female). The parents were killed after 24 hours. The number of GFP-expressing and non-expressing animals was counted after another 24 hours at 25°*C*. If a plate contained few or only a very small number of animals without GFP expression, a single GFP-expressing male and a single GFP-expressing female were selected and allowed to mate on a fresh plate with 10 replicates, and the steps were repeated once. An absence of cross progeny showing no GFP expression in two consecutive generations was assumed to be a successful candidate for homozygous introgression. For a single introgression, if the above steps were attempted five times without obtaining a homozygous introgression line, the introgression fragment was deemed inviable as a homozygote in *C. nigoni*.

### Phenotype scoring

**Male sterility**. Male sterility was determined only for those containing introgression fragment present as heterozygote or hemizygote. Introgressions that can be rendered as viable homozygote were assumed as male fertile and viable. Male sterility was assumed when no progeny were found in a cross between seven GFP-expressing heterozygous or hemizygous males and five *C. nigoni* (JU1421) L4 females in three replicates but progeny were readily found in the cross between seven *C. nigoni* (JU1421) males and five GFP-expressing L4 females picked from the same brood as the GFP-expressing males.

**Male inviability**. Male inviability was defined as the absence of GFP-expressing males (L4 or adult) during a cross between five GFP-expressing heterozygous females and seven *C. nigoni* (JU1421) males in three replicates.

**Embryonic lethality**. For homozygous introgression animals, 10 gravid young adults were allowed to lay eggs for 5 hours at 25°C before killing all of the parents. Five replications were performed. The total number of eggs was counted immediately after removal of the parents. The number of eggs left on the plates was re-counted after overnight (ON) incubation. Embryonic lethality was calculated as the percentage of un-hatched eggs out of the total number of eggs laid. For heterozygous introgressions, embryonic lethality was scored for the mating progeny between GFP-expressing males and GFP-expressing females. 25% of the crossing progeny from the parents both carrying a recessive autosome-linked heterozygous introgression is expected to be inviable if the introgression produces fully penetrant embryonic lethality. Embryos from *C. nigoni* inbreeding adults (JU1421) were included as control.

**Larval arrest**. The number of hatched L1 larvae in the above experiments was counted at the same time as the un-hatched eggs after ON incubation. The number of adults on each plate was counted after incubation of the eggs at 25°C for another 48 hours. Five replications were performed in parallel. The larval arrest ratio was defined as the difference between the number of larvae and adults divided by the number of hatched L1 larvae. Inviability referred to either embryonic lethality or larval arrest. Larvae from *C. nigoni* inbreeding adults (JU1421) were included as control.

**Brood size**. For homozygous introgressions, a single young male and a single L4 female were mated on a single seeded NGM plate for 12 hours at 25°C with 10 replicates. The gravid females were transferred to a fresh plate every 12 hours for 72 consecutive hours at 25°C. The number of eggs on each plate was counted immediately after the transfer. Only eggs laid by the parent that survived the entire 72 hours were counted. For heterozygous introgressions, a single GFP-expressing young adult male and a single GFP-expressing L4 female were mated on individual plates in 10 replicates. The remaining steps were the same as those for homozygous introgressions. The brood size was defined as the total number of eggs laid during the 72 hours. The brood size from *C. nigoni* (JU1421) young adults was similarly counted as the control.

**Percentage of GFP-expressing and non-GFP-expressing male progeny**. For homozygous introgression lines, 10 young gravid adults were allowed to lay eggs on an NGM plate for three hours followed by removal of the parents. The plate was set up for five replications. The eggs were incubated at 25°C for 72 hours. The numbers of males and females were counted respectively. As all of the progeny expressed GFP, the percentage of males out of the total progeny was calculated. For heterozygous introgressions (homozygous likely inviable) on the autosome, 10 GFP-expressing young males and 20 GFP-expressing L4 females were mated on a single plate with five replicates. Ten female adults with successful mating as judged by the presence of a mating plug were transferred onto a fresh plate after overnight incubation at 25°C and allowed to lay eggs for three hours followed by removal of the parents. The eggs were incubated at 25°C for another two days. The experiment was replicated five times. The numbers of GFP-expressing and non-GFP-expressing males and females were counted respectively. The percentages of GFP-expressing animals and GFP-expressing males were calculated by dividing their numbers with the total number of progeny. For X-linked male sterile or inviable introgression, the percentage of GFP-expressing progeny was similarly calculated, except that 10 heterozygous GFP females were crossed with 20 *C. nigoni* males. The overall ratio of GFP-expressing progeny was scored as the percentage of total GFP-expressing progeny out of total crossing progeny.

### Statistical analysis

To determine the statistical significances between mean percentages of Emb, Lva and Brood size from both homozygous and heterozygous introgressions and those of the control, which is inbreeding crossing between *C. nigoni* males and females (JU1421), one-way ANOVA was performed among all groups of each phenotype to evaluate whether there were any significant differences between groups. Tukey's Honestly Significant Difference (Tukey’s HSD) test was then used as a post hoc test to calculate the significance of mean between introgression and control for each phenotypic category.

To determine whether there is any significant bias on sex segregation or sex-specific segregation of GFP, chi-squared test (X^2^) test was performed for the male percentage of homozygous introgression with expected percentage of 50. X^2^ test was also performed to examine sex-specific segregation of GFP for heterozygous introgressions, including percentage of autosome and X-linked GFP-expressing and non-GFP expressing male progeny out of total GFP expressing progeny with an expected percentage of 50%; and autosome and X-linked overall percentage of GFP -expressing progeny with an expected percentage of 75% and 50% respectively if an X-linked introgression male is viable or 75% and 33.3% respectively if an X-linked introgression male is inviable.

## Supporting Information

S1 Fig Strategies for generation of stable transgenic reporters in *C. briggsae*. (A) Flowchart for generating stable transgenic strains in *C. briggsae*. (B) A fluorescence micrograph of reporter expression under a stereo microscope.
(TIF)

S2 Fig Validation of PCR mapping results by NGS. Shown are the validations for two homozygous introgression lines ZZY10296 (A-C) and ZZY10291 (D-F) located on chromosome V and II respectively. Drawn are plots of the read coverage (Y axis) against *C. briggsae* chromosomal coordinates (X axis). Reads in A & D or B & E were derived from genomic DNAs of *C. briggsae* and *C. nigoni* respectively and reads in C & F from ZZY10296 (C) or ZZY10291 (F) respectively. For introgression ZZY10296 (C), it was mapped into a genomic interval of 7.75 Mb, i.e., chromosome V: 0–7.75 Mb by single-worm PCR (S1 and S3 Table). Note the read coverage of the introgression was significantly deeper from 0 to 7.10 Mb (shaded in grey), the interval of which is consistent with that obtained by the PCR albeit a bit smaller in size, which is likely owing to the low primer density. In addition, the patterns of read fold enrichment for the introgression region were more similar to those of *C. briggsae* (A) than to those of *C. nigoni* (B). The remaining part of ZZY10296 shows the similar enrichment patterns as those of *C. nigoni* (B). For introgression ZZY10291, it was mapped into a genomic interval of 0.45 Mbs, i.e., chromosome II: 6.49–6.94 Mb by the single-worm PCR (S1 and S3 Table). The NGS mapping results agree partially with that of the PCR-based genotyping and have a higher resolution (S3 Table), because a second chromosomal region at the very end of the same chromosome also shows an unusual high coverage of sequencing reads as indicated with an arrow head (shaded in green). Read coverages were plotted not in the same scale.
(TIF)

S3 Fig Example of potential assembly errors in *C. briggsae* genome (cb4) revealed during mapping of introgressions. (A) Shown were the names and positions of five pairs of primers used for genotyping introgressions of ZZY10353 and ZZY10320. Names of the remaining genotyping primers were omitted for simplicity. The chromosomal position of primer X-4 was originally located between the primers X-3.5 and X-4.5 on the X chromosome based on “cb3” assembly but relocated to an interval between primers II-12.9 and II-14 on the chromosome II based on “cb4” assembly as indicated by a tilted line. (B) Single-worm PCR results for genotyping a chromosome II-linked introgression ZZY10353 after 15 generations of backcross. The three primers, II-12.9, X-4 and II-14 showed expected amplifications with *C. briggsae* animal as a template but no amplification with *C. nigoni* (JU1421) as a template. However, the primer X-4 did not show amplification as expected from “cb4” assembly whereas it’s two flanking ones did show amplification as expected. (C) Single-worm PCR results for genotyping of an X chromosome-linked introgression ZZY10320 after 15 generations of backcross. The primer X-4 showed amplification as expected from the “cb3” assembly, which supported its position between the primer X-3.5 and X-4.5 on the X chromosome. (D) A diagram showing PCR amplification results with the primers indicated on the top using an animal carrying introgression ZZY10318 as a template. Note the PCR result with primer II-7.5 is inconsistent with those from the remaining primers, suggesting the genomic region covering the primer may belong to somewhere else in the genome.
(TIF)

S4 Fig Fertility data of the animals carrying a homozygous introgression support the rule of large X effect. (A) The X chromosomal introgressions (depicted as red bars in scale with the host chromosome in the left panel) appear to have a larger effect on fertility (right panel) than that of the autosomal introgressions. Brood sizes (depicted in horizontal line) for the control (JU1421), autosomal and X chromosomal introgressions are differentially colored in green, blue and cyan bars respectively with strain names indicated. Chromosome numbers are indicated on the top. (B) and (C) Boxplot of the brood sizes and introgression sizes (Mb) for the autosomal or the X chromosomal introgressions used in panel A respectively.
(TIF)

S5 Fig Boxplot of the sizes (Mb) for heterozygous introgressions of autosomal or X chromosomal origin. Only those used in scoring of HI phenotypes as shown in Fig. 6 were included for size calculation.
(TIF)

S6 Fig Venn diagram showing the redundancy between the introgressions that were subjected to test of homozygous viability and those used for HI phenotypic scoring. Strain names unique for each set are indicated. Number of the common introgressions used in both was indicated in the overlapping part.
(TIF)

S7 Fig Sex ratio, larval arrest (Lva) and embryonic lethality (Emb) for the homozygous introgressions. Chromosomes and introgressions were depicted in the same way as that in S4 Fig. Note that the effects of the X chromosomal introgression (blue) were comparable to those of the autosomal introgressions (cyan).
(TIF)

S8 Fig Defective spermatogenesis in a sterile male carrying an introgression. Shown are germline nuclei stained with DAPI from a male adult of *C. nigoni* (A), a male adult of sterile introgression line ZZY10307 (B) (Fig. 7) and a post-mating female adult of *C. nigoni* crossed with the ZZY10307 male (C) respectively. Germline and partial body parts are indicated. Note the dense sperm nuclei in *C. nigoni* male (A) versus the sparse sperm nuclei in the sterile male (B), suggesting defective spermatogenesis in the latter. A clear mating plug in *C. nigoni* female (C) indicates that the sterility is not caused by the defect in mating.
(TIF)

S1 Table Detailed information on the transgenic and introgression strains. (XLSX)

S2 Table List of the updated information for the genotyping primers based on “cb4” version of *C. briggsae* genome assembly. (XLSX)

S3 Table Comparison of mapping results between PCR-based and NGS-based methods. (XLSX)

S4 Table Details of phenotypic scoring of homozygous introgressions. (XLSX)

S5 Table Details of phenotypic scoring of heterozygous introgressions. (XLSX)
