# Supplementary figures and images for: A Genome-Wide Hybrid Incompatibility Landscape between Caenorhabditis briggsae and C. nigoni
Source: PLoS Genet. 2015 Feb 18;11(2):e1004993. doi: 10.1371/journal.pgen.1004993 (PMC4334894; doi:10.1371/journal.pgen.1004993)

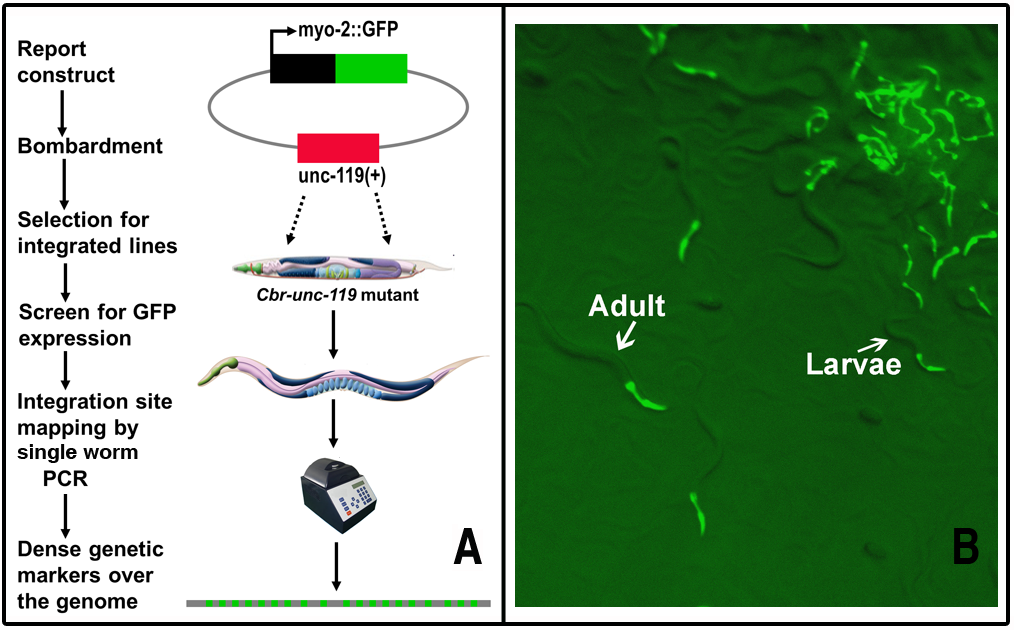

Supplement: S1 Fig — (A) Flowchart for generating stable transgenic strains in C. briggsae. (B) A fluorescence micrograph of reporter expression under a stereo microscope. (TIF) [file pgen.1004993.s001.tif]

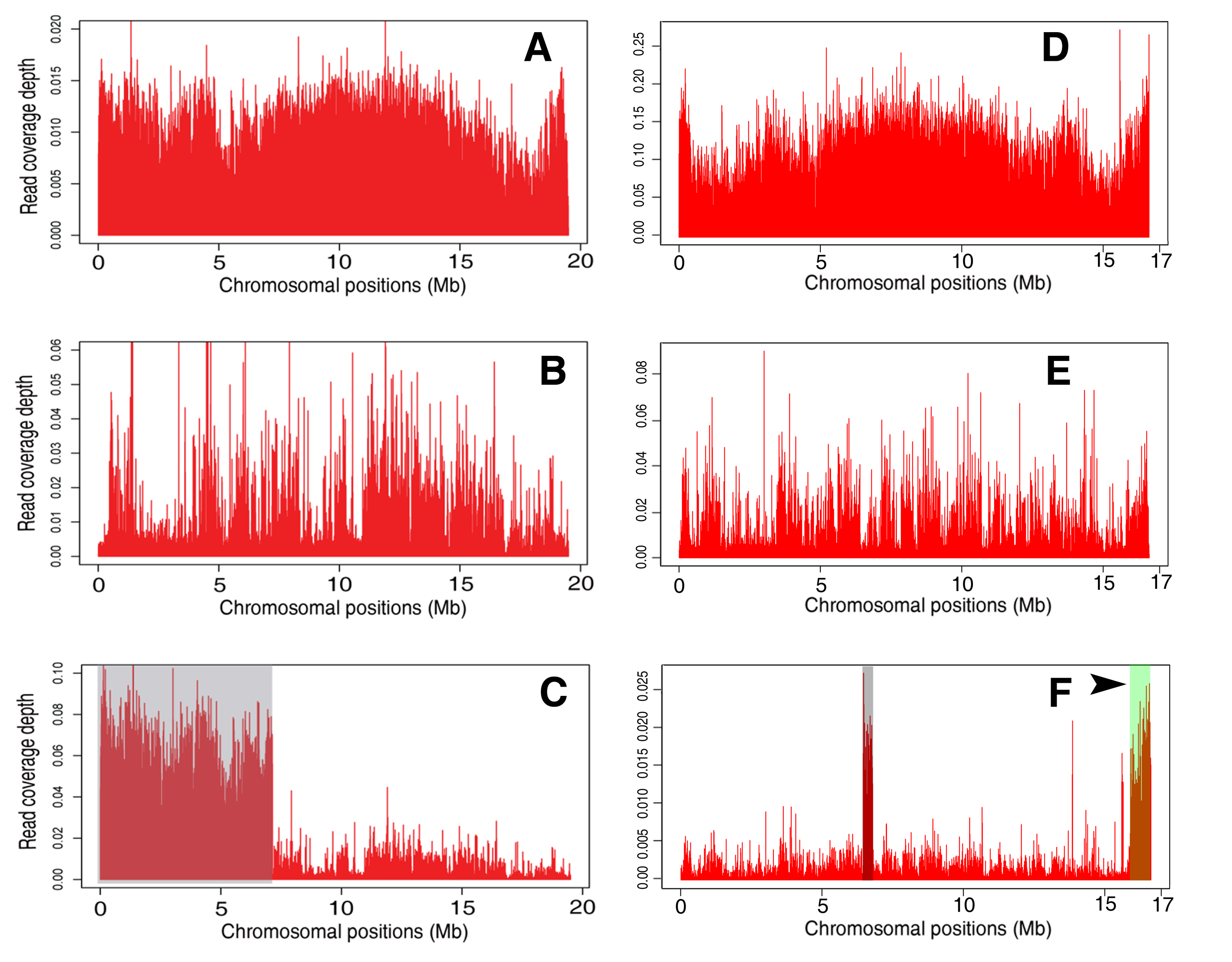

Supplement: S2 Fig — Shown are the validations for two homozygous introgression lines ZZY10296 (A-C) and ZZY10291 (D-F) located on chromosome V and II respectively. Drawn are plots of the read coverage (Y axis) against C. briggsae chromosomal coordinates (X axis). Reads in A & D or B & E were derived from genomic DNAs of C. briggsae and C. nigoni respectively and reads in C & F from ZZY10296 (C) or ZZY10291 (F) respectively. For introgression ZZY10296 (C), it was mapped into a genomic interval of 7.75 Mb, i.e., chromosome V: 0–7.75 Mb by single-worm PCR (S1 and S3 Table). Note the read coverage of the introgression was significantly deeper from 0 to 7.10 Mb (shaded in grey), the interval of which is consistent with that obtained by the PCR albeit a bit smaller in size, which is likely owing to the low primer density. In addition, the patterns of read fold enrichment for the introgression region were more similar to those of C. briggsae (A) than to those of C. nigoni (B). The remaining part of ZZY10296 shows the similar enrichment patterns as those of C. nigoni (B). For introgression ZZY10291, it was mapped into a genomic interval of 0.45 Mbs, i.e., chromosome II: 6.49–6.94 Mb by the single-worm PCR (S1 and S3 Table). The NGS mapping results agree partially with that of the PCR-based genotyping and have a higher resolution (S3 Table), because a second chromosomal region at the very end of the same chromosome also shows an unusual high coverage of sequencing reads as indicated with an arrow head (shaded in green). Read coverages were plotted not in the same scale. (TIF) [file pgen.1004993.s002.tif]

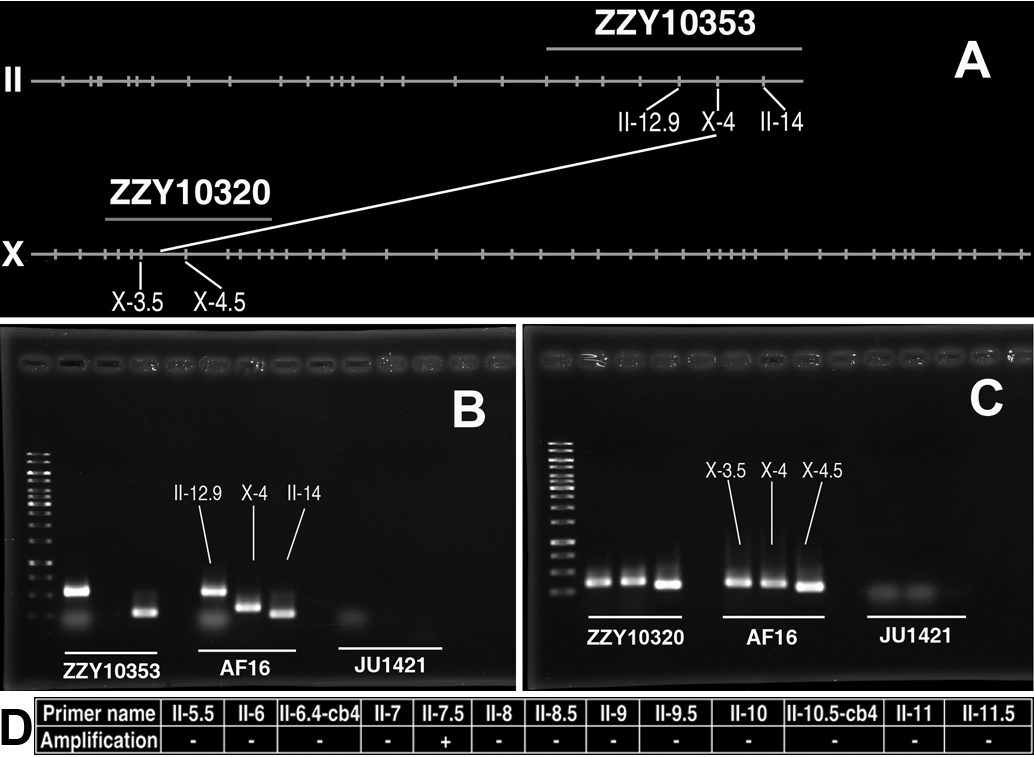

Supplement: S3 Fig — (A) Shown were the names and positions of five pairs of primers used for genotyping introgressions of ZZY10353 and ZZY10320. Names of the remaining genotyping primers were omitted for simplicity. The chromosomal position of primer X-4 was originally located between the primers X-3.5 and X-4.5 on the X chromosome based on “cb3” assembly but relocated to an interval between primers II-12.9 and II-14 on the chromosome II based on “cb4” assembly as indicated by a tilted line. (B) Single-worm PCR results for genotyping a chromosome II-linked introgression ZZY10353 after 15 generations of backcross. The three primers, II-12.9, X-4 and II-14 showed expected amplifications with C. briggsae animal as a template but no amplification with C. nigoni (JU1421) as a template. However, the primer X-4 did not show amplification as expected from “cb4” assembly whereas it’s two flanking ones did show amplification as expected. (C) Single-worm PCR results for genotyping of an X chromosome-linked introgression ZZY10320 after 15 generations of backcross. The primer X-4 showed amplification as expected from the “cb3” assembly, which supported its position between the primer X-3.5 and X-4.5 on the X chromosome. (D) A diagram showing PCR amplification results with the primers indicated on the top using an animal carrying introgression ZZY10318 as a template. Note the PCR result with primer II-7.5 is inconsistent with those from the remaining primers, suggesting the genomic region covering the primer may belong to somewhere else in the genome. (TIF) [file pgen.1004993.s003.tif]

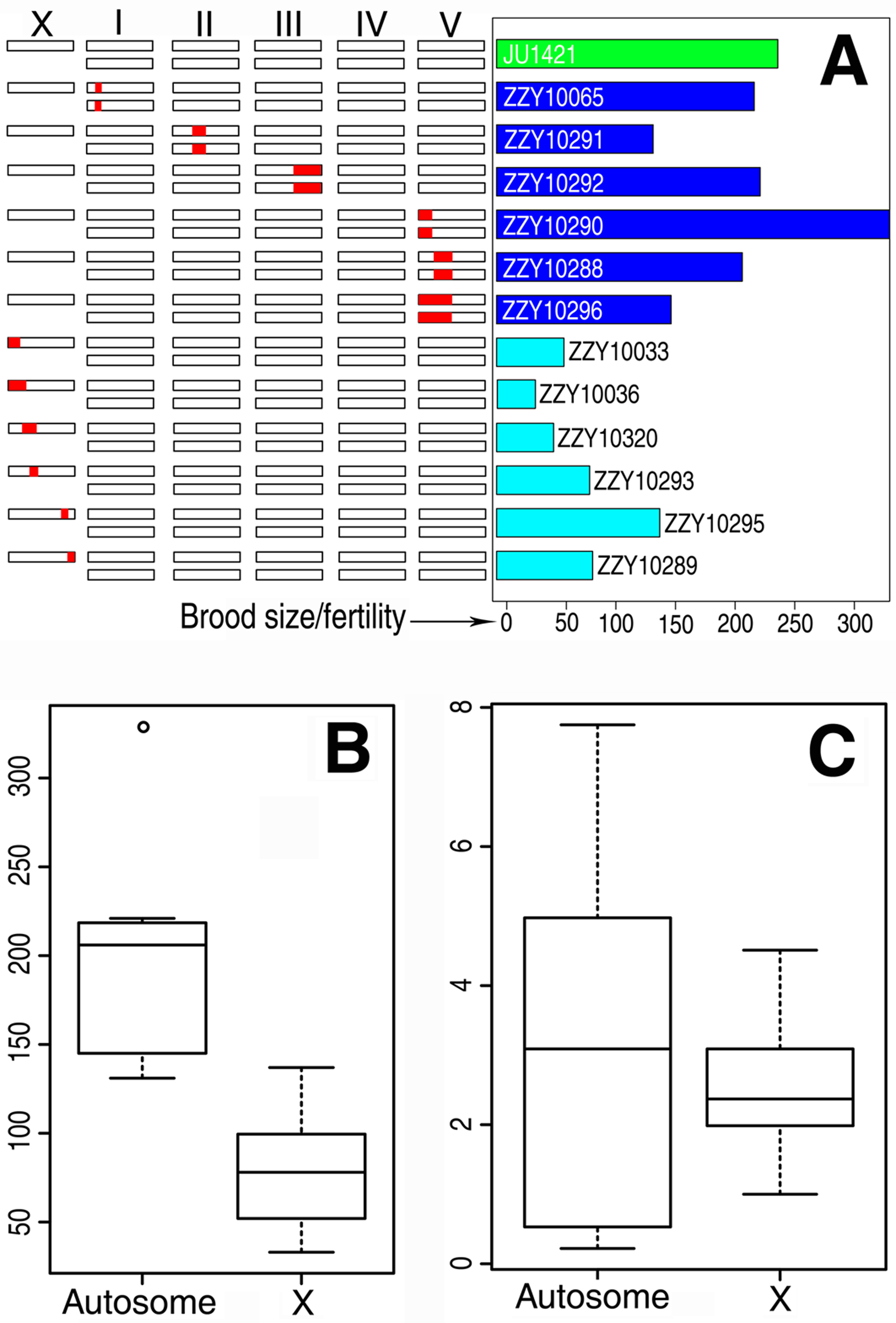

Supplement: S4 Fig — (A) The X chromosomal introgressions (depicted as red bars in scale with the host chromosome in the left panel) appear to have a larger effect on fertility (right panel) than that of the autosomal introgressions. Brood sizes (depicted in horizontal line) for the control (JU1421), autosomal and X chromosomal introgressions are differentially colored in green, blue and cyan bars respectively with strain names indicated. Chromosome numbers are indicated on the top. (B) and (C) Boxplot of the brood sizes and introgression sizes (Mb) for the autosomal or the X chromosomal introgressions used in panel A respectively. (TIF) [file pgen.1004993.s004.tif]

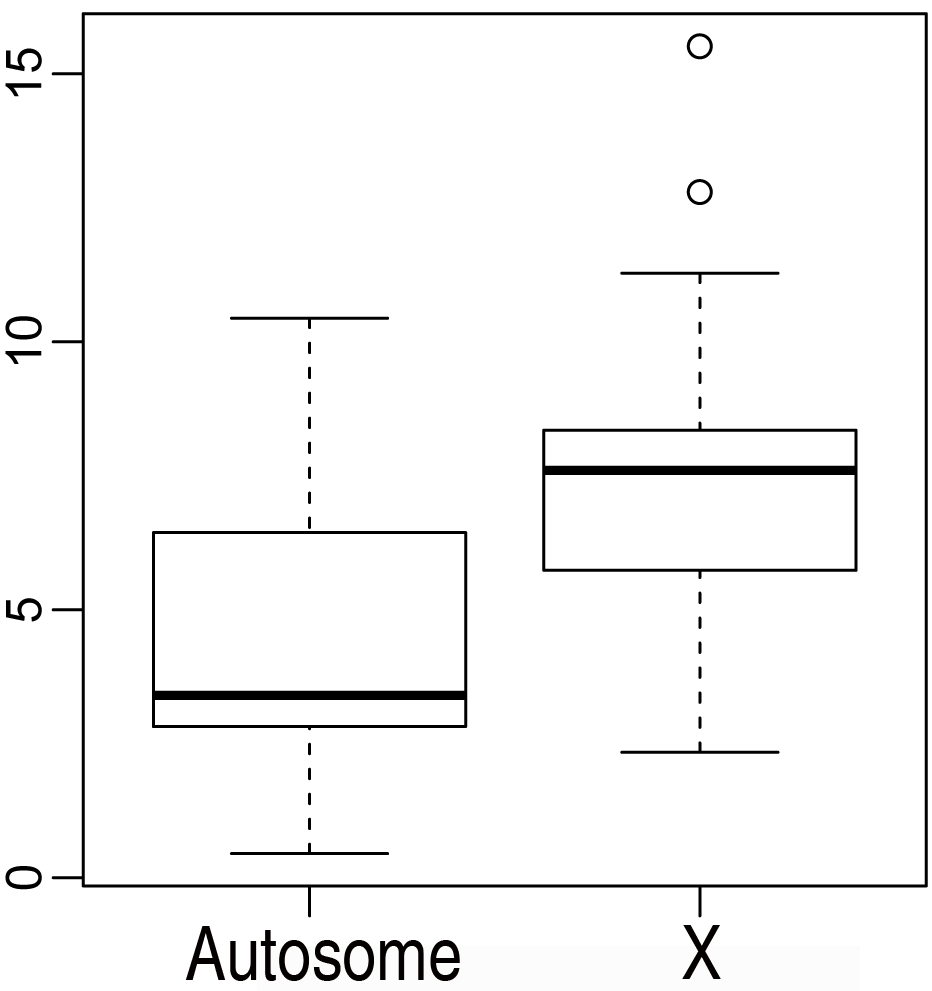

Supplement: S5 Fig — Only those used in scoring of HI phenotypes as shown in Fig. 6 were included for size calculation. (TIF) [file pgen.1004993.s005.tif]

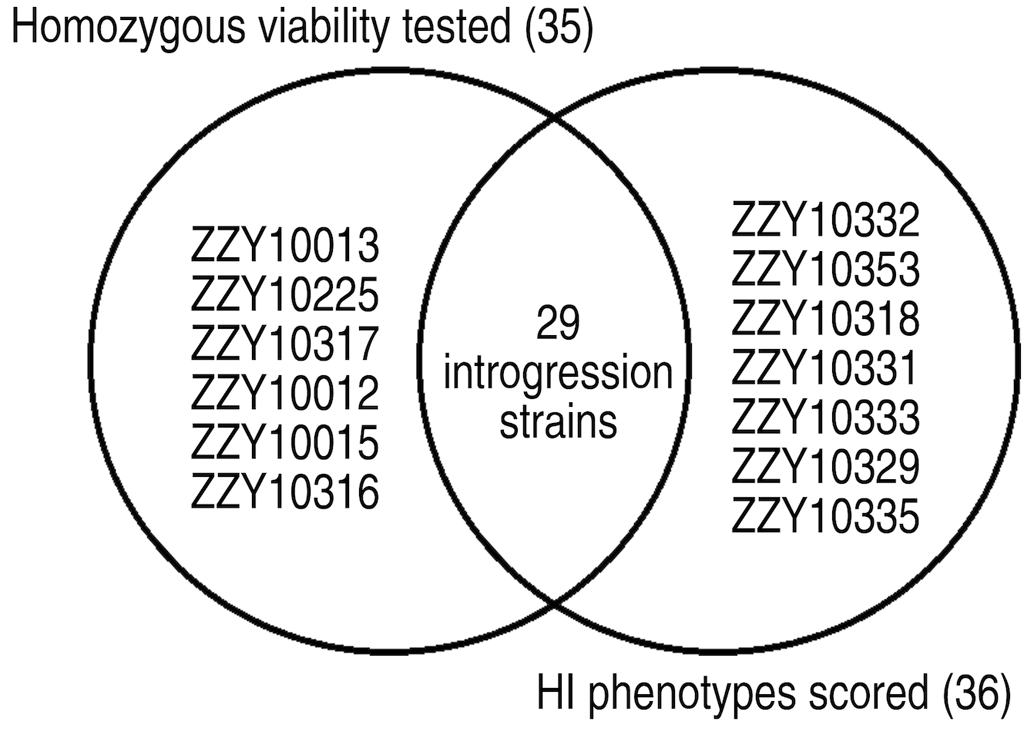

Supplement: S6 Fig — Strain names unique for each set are indicated. Number of the common introgressions used in both was indicated in the overlapping part. (TIF) [file pgen.1004993.s006.tif]

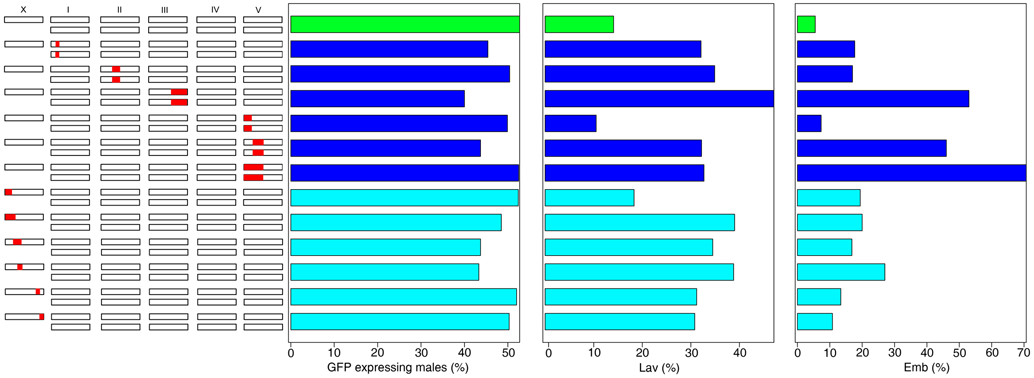

Supplement: S7 Fig — Chromosomes and introgressions were depicted in the same way as that in S4 Fig. Note that the effects of the X chromosomal introgression (blue) were comparable to those of the autosomal introgressions (cyan). (TIF) [file pgen.1004993.s007.tif]

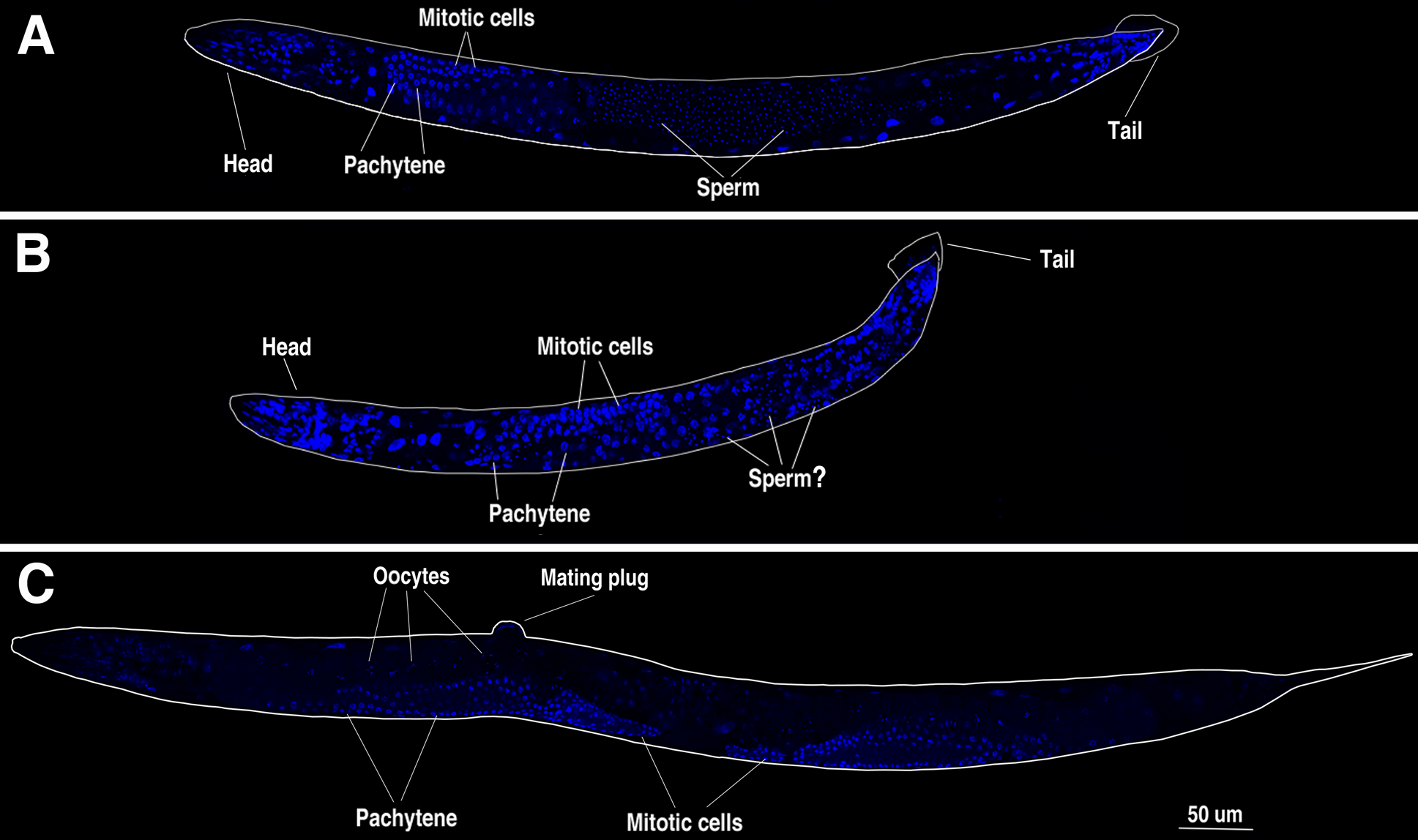

Supplement: S8 Fig — Shown are germline nuclei stained with DAPI from a male adult of C. nigoni (A), a male adult of sterile introgression line ZZY10307 (B) (Fig. 7) and a post-mating female adult of C. nigoni crossed with the ZZY10307 male (C) respectively. Germline and partial body parts are indicated. Note the dense sperm nuclei in C. nigoni male (A) versus the sparse sperm nuclei in the sterile male (B), suggesting defective spermatogenesis in the latter. A clear mating plug in C. nigoni female (C) indicates that the sterility is not caused by the defect in mating. (TIF) [file pgen.1004993.s008.tif]
